# Structural Diversity and Stability of Organic–Inorganic Hybrid Quinuclidine-Based Metal Bromides

**DOI:** 10.1021/acs.inorgchem.5c00039

**Published:** 2025-04-11

**Authors:** Ewelina Jach, Dorota A. Kowalska, Monika Trzebiatowska, Wojciech Medycki, Adam Ostrowski, Waldemar Bednarski, Marek A. Gusowski, Piotr Staniorowski, Adam Bartosiewicz, Urszula Dieu, Agnieszka Ciżman

**Affiliations:** † Department of Experimental Physics, Wrocław University of Science and Technology, 27 Wybrzeże Wyspiańskiego, Wrocław 50-370, Poland; ‡ Institute of Low Temperature and Structure Research, Polish Academy of Sciences, Okólna 2, Wrocław 50-422, Poland; § Institute of Molecular Physics, Polish Academy of Science, M. Smoluchowskiego 17, Poznań 60-179, Poland; ∥ Institute of Experimental Physics, University of Wrocław, Pl. M. Borna 9, Wrocław 50-204, Poland

## Abstract

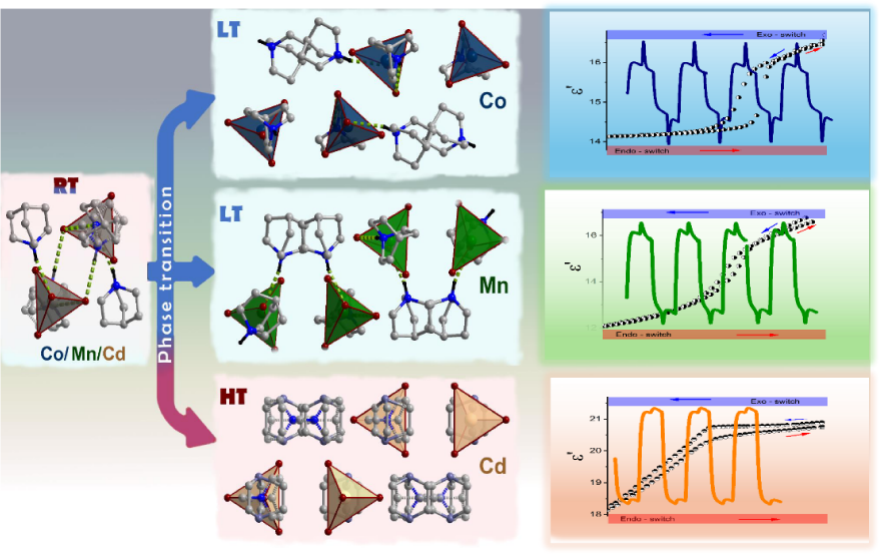

Organic–inorganic hybrid compounds based on quinuclidinium and metal bromides, (C_7_H_14_N)_2_*M*Br_4_ (*M* = Co, Mn, Cd), have been synthesized. Differential scanning calorimetry measurements indicate that all compounds undergo a reversible phase transition at 251 K (Co), 205 K (Mn), and 363 K (Cd) upon heating. The respective temperature dependences of the dielectric permittivity reveal anomalies, confirming the occurrence of phase transitions. Although the crystals are isostructural at room temperature, as confirmed by X-ray diffraction data, the mechanism of the phase transitions varies in each compound. The main driving force is the reorientation of quinuclidinium, resulting in the rearrangement of hydrogen bonds. Satisfactory dielectric and thermal stability properties of these materials have been demonstrated, highlighting their potential for applications in temperature sensors and switch devices.

## Abstract 2

Organic−inorganic hybrid compounds based on quinuclidinium and metal bromides, (C_7_H_14_N)_2_*M*Br_4_ (*M* = Co, Mn, Cd), have been synthesized.

## Introduction

The exploration of hybrid organic–inorganic compounds (HOICs) represents a vibrant and rapidly evolving domain in materials science, offering a fascinating confluence of organic and inorganic chemistries. These hybrids, known for their unique amalgamation of properties derived from both constituents, have emerged as a versatile class of materials with vast potential in various technological applications.^1−3^ It is this versatility of HOICs, encompassing electrical, optical, and magnetic properties, that contributes to their growing importance in potential technological applications. A crucial aspect of HOICs is their ability to exhibit switchable dielectric properties, ferroelectric behaviors, and multiferroic phenomena, largely dictated by the dynamics of hydrogen bonding within their structures. These properties hold immense significance in the development of advanced electrical and electronic devices. Materials exhibiting switchable dielectric behavior, oscillating between high and low dielectric states, are particularly sought after for their potential in applications such as phase shifters, varactors, data communication systems, and rewritable optical data storage. At the microscopic level, the dielectric response in HOICs can be attributed to complex mechanisms, such as dipolar reorientation, ionic displacement, and electronic polarization. The interplay of these mechanisms, especially in temperature ranges above and below phase transition thresholds, defines the dielectric state of the material. High-temperature regimes favor dipolar reorientation, leading to a high-dielectric “ON” state, while lower temperatures result in the freezing of these reorientations, leading to a low-dielectric “OFF” state. Consequently, the ordering and dynamics of ions or polar molecules within HOICs are pivotal in modulating their dielectric properties, offering a tunable platform for various applications. The design and synthesis of HOICs often focus on leveraging reversible structural phase transitions to achieve the desired functional properties. These transitions are primarily facilitated by order–disorder transformations of polar organic cations and subtle displacements within the inorganic frameworks. From the point of view of electronic properties, lead-based halide perovskites of the *ABX*_3_ formula, where *B* = Pb and *X* = I, Br, or Cl, have been mostly analyzed.^4−7^ Such hybrids with *X* = Br have a higher bandgap and light emission at shorter wavelengths, while hybrids with iodine have smaller bandgaps and light emission at longer wavelengths. Mainly due to the larger electronegativity of Cl compared to Br or I, the band absorption peaks for *X* = Cl shift toward higher energy.^8^ It has been shown that the substitution of Br with Cl atoms in CsPbBr_3_ increases the bandgap to approximately 2.7 eV.^8−10^ This indicates that the optoelectronic properties of halide perovskites can be effectively modified by adjusting the compositions of these materials. Generally, modification of the halide anion affects the bond distance of *X*–Pb–*X* and is one of the methods of tuning the energy gap.^10−12^ It has been shown that in lead halide perovskites with the methylammonium cation, the bond Pb–Pb intraspecies transition, with some impact of the proportion of *X*–*X* play essential role in the optical properties. Moreover, it has been shown that the *A*-site (organic) cations, e.g., methylammonium cations, do not directly contribute to the valence and conduction bands. However, experimental results suggest that changes in *A*-site (organic) cations can adjust energy levels to a certain extent. Nevertheless, by selectively exchanging and combining the *B* cations and *X* anions, the bandgap and emission properties of a perovskite can be tuned.^11^ It has been pointed out that the substitution of cations by organic amines multiplies the crystal structures and modifies them.^12^

For instance, spherical molecules like dabco (1,4-diazabicyclo[2.2.2]octane)^13^ or quinuclidinium (Q)^14,15^ have demonstrated intriguing physicochemical properties and structural phase transitions through dynamic rotation or reorientation. The unstable nature of hydrogen bonds in these materials significantly contributes to their structural versatility, affecting their optical, electrical, and magnetic properties. Recent advancements have further highlighted the potential of HOICs with spherical organic cations as promising candidates for switchable dielectric materials. Temperature-induced dielectric permittivity changes, combined with order–disorder transformations, pave the way for materials exhibiting unique electric, magnetic, and optoelectronic properties. For instance, quinuclidinium-based metal chlorides^14,15^ have shown switchable properties stemming from the rotational dynamics of Q ions, leading to reversible phase transitions. Such materials are not only of academic interest but also hold substantial promise for practical applications in next-generation smart materials and devices. The development of HOICs with tailored properties necessitates a holistic approach, encompassing the synthesis of new compounds, detailed structural characterization, and an understanding of their thermal, dielectric, and magnetic properties. Techniques, such as differential scanning calorimetry, single-crystal X-ray diffraction, Raman spectroscopy, and dielectric constant measurements, are instrumental in unraveling the complex interplay between the organic and inorganic components in these hybrids. A systematic investigation of these materials aims to unlock their full potential, contributing to the advancement of materials science and opening new avenues in technological development.

## Materials and Methods

### Synthesis

#### Materials

Cadmium bromide tetrahydrate (CdBr_2_·4H_2_O 98%, pure, Thermo Scientific Chemicals); quinuclidine (1-azabicyclo[2.2.2]octane, 98%, AmBeed); hydrobromic acid (HBr, 48% in water, Fluorochem); methanol (99.85+%, for analysis, Fisher); acetonitrile (HPLC super gradient, Chempur). All chemicals were used as received.

##### Synthesis of (Quinuclidine·HBr)_2_CdBr_2_

To obtain the (quinuclidine·HBr)_2_CdBr_2_ crystals, a molar ratio of 2:1 of quinuclidine and CdBr_2_·4H_2_O was taken. Quinuclidine (2 mmol, 222 mg) was dissolved in a 30 mL methanol/acetonitrile solution (1:1 v/v) whose task is to dissipate temperature during the conversion of quinuclidine into QBr salt, and it is a reaction environment. HBr (ca. 2 mL) was added dropwise until pH = ∼1 in the quinuclidine methanol/acetonitrile solution. Separately, CdBr_2_·4H_2_O (1 mmol, 344 mg) was dissolved in 20 mL methanol/acetonitrile (1:1 v/v) with the addition of a few drops of HBr. The solution of CdBr_2_ was added, while stirring, to the solution of quinuclidine·HBr. After a homogeneous transparent liquid was obtained, the solution was left undisturbed to slowly evaporate over several days. The crystals of (quinuclidine*·*HBr)_2_CdBr_2_ salt (550 mg, 84% yield based on CdBr_2_) were then collected in the form of regularly sized transparent (quinuclidine·HBr)_2_CdBr_2_ crystals and utilized without further purification.

##### Synthesis of (Quinuclidine·HBr)_2_CoBr_2_

To obtain the (quinuclidine*·*HBr)_2_CoBr_2_ crystals, a molar ratio of 2:1 of quinuclidine and CoBr_2_ was taken. Quinuclidine (2 mmol, 222 mg) was dissolved in 30 mL of a methanol/acetonitrile solution (1:1 v/v). HBr (ca. 2 mL) was added dropwise until pH = ∼1 to the quinuclidine in the methanol/acetonitrile solution. Separately, CoBr_2_ (1 mmol, 219 mg) was dissolved in 20 mL of methanol/acetonitrile (1:1 v/v) with the addition of a few drops of HBr. The solution of CoBr_2_ was added, while being stirred, to the solution of quinuclidine·HBr. After obtaining a homogeneous dark blue transparent liquid, the solution was left undisturbed to slowly evaporate over several days. The crystals of (quinuclidine*·*HBr)_2_CoBr_2_ salt (530 mg, 88% yield based on CoBr_2_) were then collected in the form of regular-sized dark green transparent (quinuclidine·HBr)_2_CoBr_2_ crystals and utilized without further purification.

##### Synthesis of (Quinuclidine·HBr)_2_MnBr_2_

To obtain the (quinuclidine*·*HBr)_2_MnBr_2_ crystals, a molar ratio of 2:1 of quinuclidine and MnBr_2_ was taken. Quinuclidine (2 mmol, 222 mg) was dissolved in a 30 mL methanol/acetonitrile solution (1:1 v/v). HBr (ca. 2 mL) was added dropwise until pH = ∼1 to the quinuclidine in the methanol/acetonitrile solution. Separately, MnBr_2_ (1 mmol, 215 mg) was dissolved in 20 mL of methanol/acetonitrile (1:1 v/v) with the addition of a few drops of HBr. The solution of MnBr_2_ was added, while stirring, to the solution of quinuclidine·HBr. After a homogeneous yellow transparent liquid was obtained, the solution was left undisturbed to slowly evaporate over several days. The crystals of (quinuclidine·HBr)_2_MnBr_2_ salt (533 mg, 89% yield based on MnBr_2_) were then collected in the form of regularly-sized transparent yellow (quinuclidine·HBr)_2_MnBr_2_ crystals and utilized without further purification.

### Thermal Properties

Thermal property measurements were performed on a DSC instrument (DSC-1, Mettler Toledo) during cooling and heating processes at a temperature change rate of 10 K/min, within the 200–420 K temperature range, under nitrogen gas.

### Structural Properties

Q_2_*M*Br_4_ crystals of good quality were chosen for diffraction measurements on an Oxford X’Calibur four-circle diffractometer using graphite-monochromated Mo *K*α (λ = 0.71073 Å) radiation. The instrument was equipped with a CCD Atlas detector, and an Oxford Cryosystem 800 series cryocooler was used to maintain nonambient temperatures. The program CrysAlis PRO^16^ was employed for data collection and reduction. Empirical absorption correction using spherical harmonics, implemented in the SCALE3ABSPACK scaling algorithm, as well as numerical absorption correction based on Gaussian integration over a multifaceted crystal model, were applied. The data at different temperature points were collected from the same crystal samples for all three presented compounds.

The crystallographic software package SHELX-2014,^17,18^ incorporated into the Olex2^19^ program, was used to solve the structures by direct methods and refine them using full-matrix least-squares methods on *F*^2^. Non-hydrogen atoms were refined anisotropically, except for the C and N atoms of disordered Q ions. The H atom parameters were constrained. The details of the crystal data, together with experimental and refinement information on phases I and II of Q_2_MBr_4_, are gathered in Table 1. In Table S1, we have placed the relevant structural information regarding phase II of Q_2_CdBr_4_ at 100 K, in which the motion of Q cations is frozen, and thus the order of all ions can be observed (see Figure S1). The crystallographic data can be obtained from the Cambridge Crystallographic Data Centre with reference numbers 2383365–2383371.

**Table 1:** Diffraction Experimental Details

	Q_2_CoBr_4_		Q_2_MnBr_4_		Q_2_CdBr_4_	
Phase	**II**	**I** (disordered)	**II**	**I** (disordered)	**II** (disordered)	**I** (disordered)
Crystal data						
Chemical formula	2(C_7_H_14_N)·CoBr_4_		2(C_7_H_14_N)·MnBr_4_		2(C_7_H_14_N)·CdBr_4_	
*M*_r_	602.95		598.96		656.42	
Crystal system, space group	Monoclinic, *P*2_1_/*n*	Monoclinic, *P*2_1_/*c*	Triclinic, *P*1̅	Monoclinic, *P*2_1_/*c*	Monoclinic, *P*2_1_/*c*	Orthorhombic, *Pmcn*
*T* (K)	100	295	100	295	295	365
*a*, *b*, *c* (Å)	9.617(3), 12.461(4), 16.716(5)	9.559(3), 17.120(5), 12.789(4)	9.488(3), 17.051(5), 12.650(4)	9.608(3), 17.187(4), 12.929(4)	9.625(3), 17.181(5), 12.997(4)	9.746(4), 17.224(6), 13.128(5)
α, β, γ (°)	90, 93.28(3), 90	90, 92.26(3), 90	91.41(3), 92.84(3), 88.87(3)	90, 91.91(3), 90	90, 91.60(3), 90	90, 90, 90
*V* (Å^3^)	1999.9(11)	2091.3(11)	2043.0(11)	2133.8 (11)	2148.4(11)	2203.7(15)
*Z*	4		4		4	
μ (mm^–1^)	8.85	8.46	8.47	8.11	8.45	8.24
Crystal size (mm)	0.31 × 0.22 × 0.19		0.44 × 0.32 × 0.28		0.28 × 0.17 × 0.14	
Data collection						
Refl. measured/unique/observed [*I* > 2σ(*I*)]	9430/9430/5667	17142/4266/2547	15816/15816/10937	12853/4038/2517	34369/4387/3370	21826/2394/1278
*R*_int_	0.086	0.029	0.046	0.026	0.026	0.031
Refinement						
*R*[*F*^2^ > 2σ(*F*^2^)], *wR*(*F*^2^), *S*	0.073, 0.197, 1.00	0.060, 0.174, 1.04	0.042, 0.100, 0.98	0.059, 0.188, 1.06	0.047, 0.129, 1.02	0.064, 0.241, 1.05
Data/parameters/restraints	9430/191/0	4266/183/30	15816/380/60	4038/183/30	4387/182/45	2394/101/32
Δρ_max_, Δρ_min_ (e Å^–3^)	2.17, –1.42	0.81, –0.75	16, –1.00	75, –0.65	92, –0.78	70, –0.57
Absolute structure	Refined as two comp. twin		Refined as two comp. twin			
Abs. struct. parameter	0.430(2)		0.372(6)			

In order to confirm the phase purity of the bulk samples, powder X-ray diffraction (PXRD) data were collected. The PXRD measurements were performed in reflection mode using a PANalytical X’Pert diffractometer with a PIXcel solid-state linear detector and using Cu *K*α (λ = 1.5418 Å) radiation (Figure S2).

### Raman and IR Spectroscopy

The temperature-dependent infrared spectra were measured in heating mode on a KBr pellet in the range of 4000–550 cm^–1^ using a Nicolet iN10 Fourier transform IR spectroscopy microscope equipped with a ZnSe-Linkam cryostat cell (THMS600) with a temperature stability of 0.1 K, a liquid nitrogen ((LN_2_)–cooled) mercury–cadmium–telluride detector, a permanently aligned 15× objective, and a 0.7 numerical aperture with the working distance set at 16 mm. The Raman spectra were measured in heating mode using a Renishaw InVia Raman spectrometer equipped with a confocal DM 2500 Leica optical microscope, a thermoelectrically cooled CCD as a detector, and a diode laser operating at 488 nm, which allowed measurements within 50–3200 cm^–1^ (for Q_2_CoBr_4_ and Q_2_CdBr_4_ samples) and an IR laser operating at 830 nm, resulting in 50–2000 cm^–1^ range for the Q_2_MnBr_4_ sample due to its high luminescence when probed with 488 or 514 nm. The spectral resolution in the IR and Raman experiments was set at 2 cm^–1^.

### Electrical Properties

The complex electric permittivity *ε=ε’* + *iε’’* measurements were conducted using Broadband Dielectric Spectroscopy (BDS) with the aid of the Novocontrol Alpha Impedance Analyzer. The measurements were performed in a frequency range from 1 Hz to 1 MHz using an amplitude of 1 V and at a temperature range of 200–420 K. The temperature was controlled by a nitrogen gas cryostat with a stability higher than 0.1 K. Pelletized samples, made of pressed polycrystalline powder with an area of approximately 5 mm^2^, were used as capacitors. Silver paste was applied to the samples’ surfaces to ensure good electrical contact.

### ^1^H NMR Spin–Lattice Relaxation Time

The ^1^H NMR measurements were made using an ELLAB TEL-Atomic PS 15 operating at ω_H_ = 25 MHz at temperatures from 84 K up to 290 K for the nonparamagnetic samples of Q_2_MnBr_4_ and Q_2_CdBr_4_ and for paramagnetic Q_2_CoBr_4_ from about 145 K up to 290 K.

Spin–lattice relaxation times, *T*_1_, were measured using a saturation sequence of π/2 pulses, followed by a variable time interval τ and a reading π/2 pulse. The second moment, *M*_2_, of the ^1^H NMR line was measured in the temperature range from 109 to 290 K using a continuous-wave ELLAB TEL-Atomic CWS 12–50 spectrometer working on protons at a frequency of 28.2 MHz. The *M*_2_ values were found by numerical integration of the absorption curve derivatives.

The magnetization was found to recover nonexponentially at all temperatures. The low temperatures of the sample were obtained by cooling using liquid nitrogen vapor and were controlled by a UNIPAN 660 temperature controller operating on a Pt 100 sensor, providing long-term temperature stability better than 1 K. The powdered samples were evacuated at room temperature and then sealed under vacuum in glass ampules. All measurements were made while heating the samples. The errors in the measurements of *T*_1_ were estimated to be about 5%.

### EPR Study

EPR measurements were performed using an X-band Bruker ELEXSYS spectrometer. The powdered sample was placed in a quartz tube within an ER 4102ST-type resonator. Temperature was stabilized by a Bruker ER4131RT temperature controller, and a thermocouple was used to measure the sample temperature.

## Results

### Phase Transition

**Figure 1:**
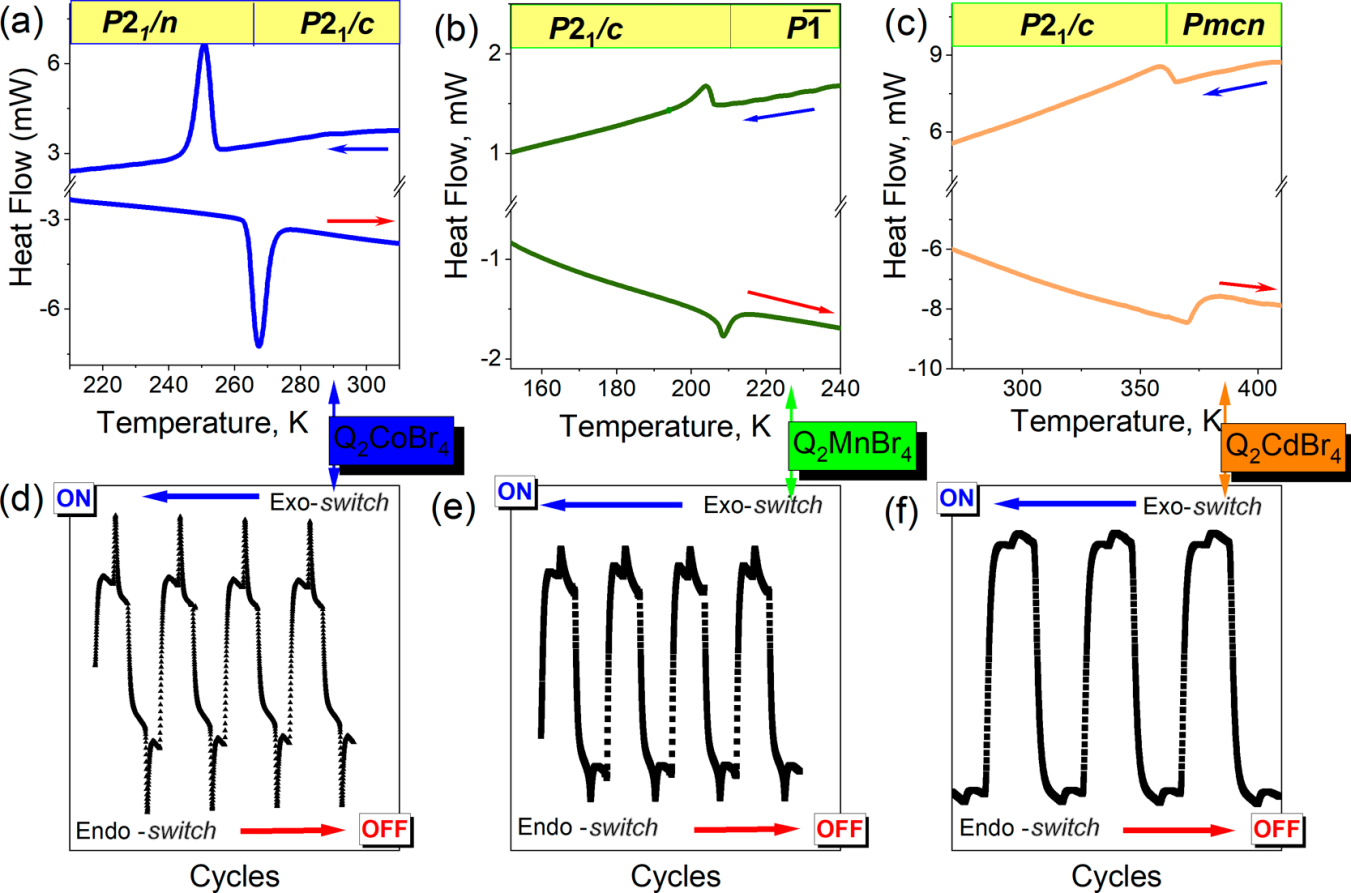
DSC analysis for Q_2_CoBr_4_ (a), Q_2_MnBr_4_ (b), Q_2_CdBr_4_ (c), and DSC cycle test of compounds Q_2_CoBr_4_ (d), Q_2_MnBr_4_ (e), and Q_2_CdBr_4_ (f) with a scan rate of 10 K·min^–1^.

Thermal analyses using differential scanning calorimetry (DSC) were employed to detect the occurrence of a phase transition. In their DSC traces (Figure 1), endo/exothermic peaks were observed at 267/251 K (Q_2_CoBr_4_), 208/205 K (Q_2_MnBr_4_), and 369/363 K (Q_2_CdBr_4_) upon heating/cooling. The Δ*S* values for the phase transitions were estimated as 10.5 (Q_2_CoBr_4_), 2.5 (Q_2_MnBr_4_), and 3.8 J mol^–1^ K^–1^ (Q_2_CdBr_4_). Given that Δ*S* = *R* ln*N*, where *R* is the gas constant and *N* is the ratio of the numbers of states in different phases, the calculated *N* values were 3.53 (Q_2_CoBr_4_), 1.35 (Q_2_MnBr_4_), and 1.58 (Q_2_CdBr_4_). The *N* values being less than two for Q_2_MnBr_4_ and Q_2_CdBr_4_ indicate a complicated phase transition. Moreover, the broad peaks with extended tails and small thermal hysteresis for these compounds suggest second-order phase transitions. For Q_2_CoBr_4_, an *N* value much greater than 2.0, combined with a large thermal hysteresis of ∼16 K, corresponds well to an order–disorder first-order phase transition. Furthermore, both the ordered phase and the phase transition mechanism of Q_2_CoBr_4_ are similar to the Q_2_CoCl_4_ analogue, where a first-order phase transition was observed.^15^

The DSC measurements were performed for several heating/cooling cycles (Figure 1 d–f). These results indicate that after a few endothermic and exothermic cycles, both the phase transition and the endothermic/exothermic values of the compounds can still persist and do not change significantly. It is commonly understood that when the temperature rises to *T*_*p*_, heat energy can be stored in latent heat form, and when the temperature drops below *T*_*p*_, this stored energy can be released.^20,21^ Taking the above into account, Q_2_CoBr_4_, Q_2_MnBr_4_, and Q_2_CdBr_4_ compounds may have some potential applications as thermal energy storage materials.

### Crystals Structure Analysis

The crystal structures of compounds Q_2_CoBr_4_ and Q_2_CdBr_4_ at room temperature are isostructural with the previously known structure of Q_2_MnCl_4_^14^. All three compounds crystallize in the centrosymmetric space group *P*2_1_/*c*.

**Figure 2:**
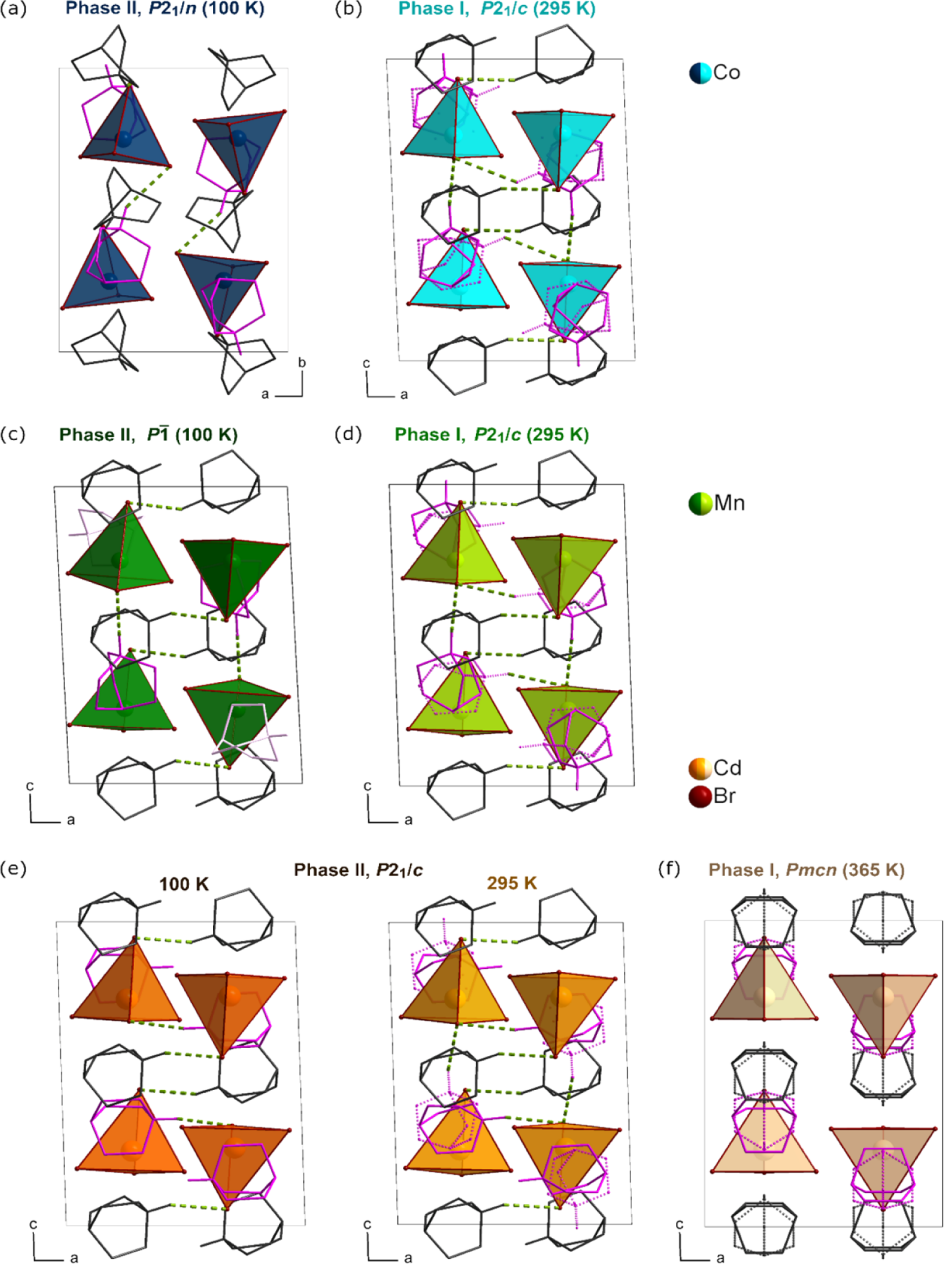
Comparison of the views at Q_2_CoBr_4_ (a,b), Q_2_MnBr_4_ (c,d), and Q_2_CdBr_4_ (e,f) in two phases. The Q ions are presented in two different colors to differentiate the Q2 cation (in pink) from the Q1 (in gray). The disordered cations are presented with dotted lines. For the sake of picture clarity, the hydrogen atoms attached to carbon atoms were removed. The N—H···Br bonds are presented as green dashed lines.

**Figure 3:**
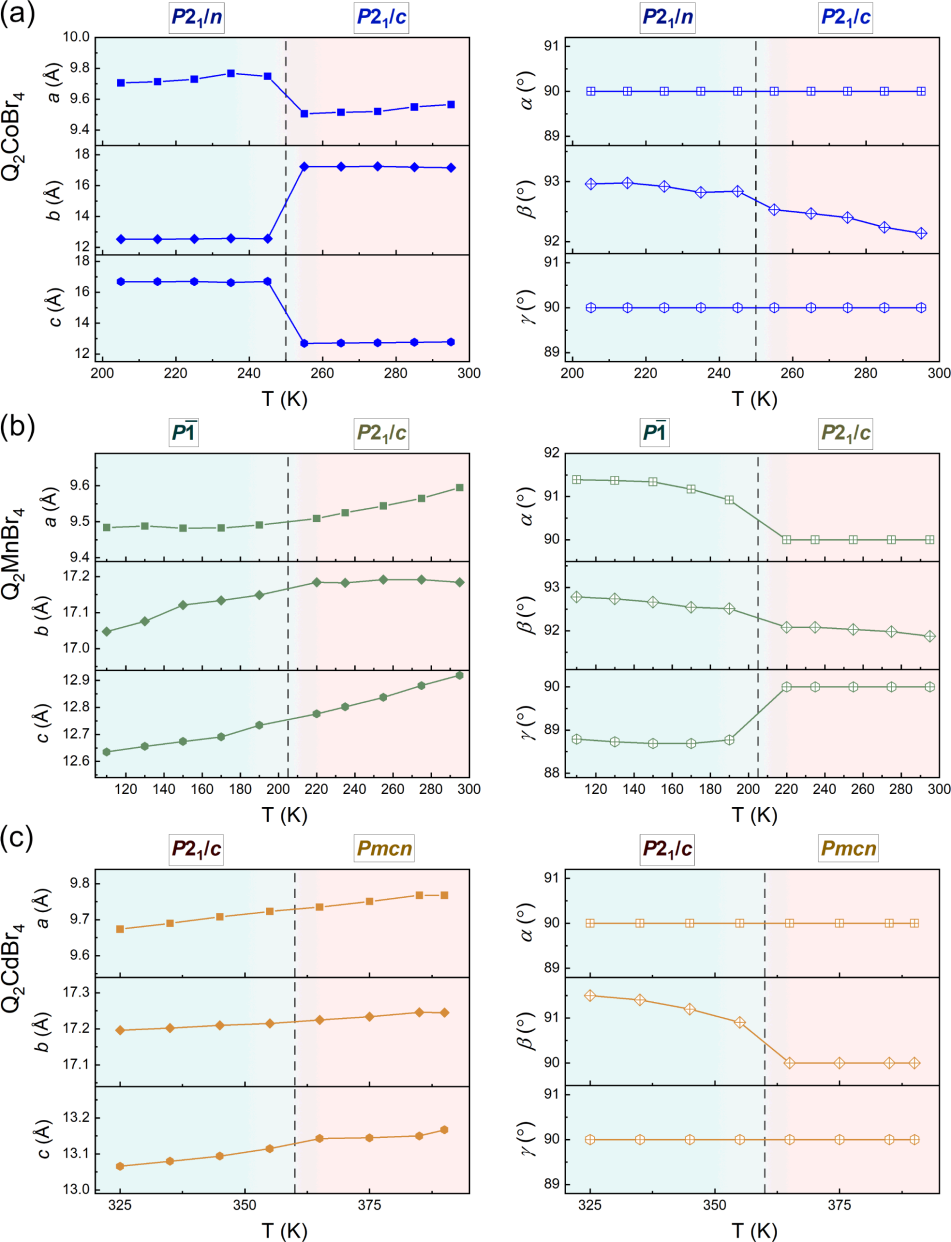
Unit cell parameters change around phase transition temperatures for Q_2_CoBr_4_ (a), Q_2_MnBr_4_ (b), and Q_2_CdBr_4_ (c).

**Figure 4:**
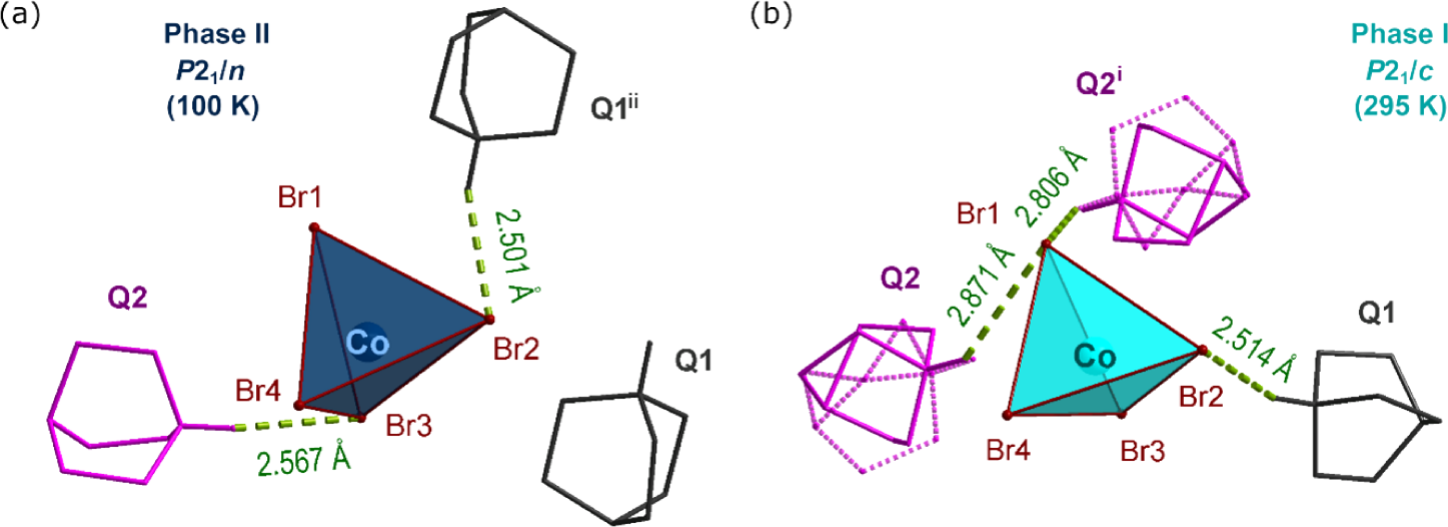
Q_2_CoBr_4_ structure showing the CoBr_4_^2–^ anion with adjacent Q cations in phase II at 100 K (a) and in phase I at 295 K (b). N—H···Br bonds are presented as green dashed lines. The disordered Q2 cations are presented with pink dotted lines. The symmetry codes: (i) −*x* + 1, −*y* + 1, −*z* + 1; (ii) −*x* + 1, −*y*, −*z* + 1.

**Figure 5:**
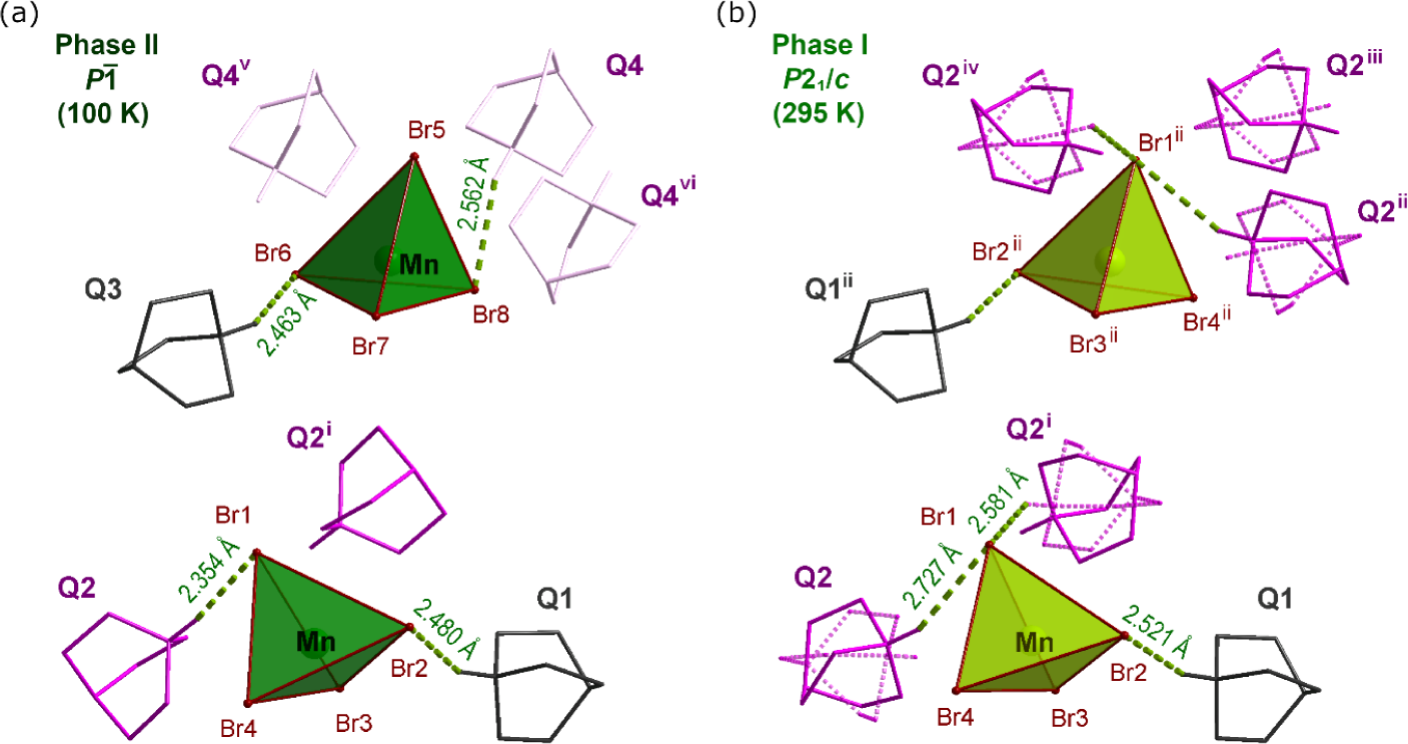
Q_2_MnBr_4_ structure showing MnBr_4_^2–^ anions with adjacent Q cations in phase II at 100 K (a) and in phase I at 295 K (b). The symmetry codes: (i) −*x* + 1, −*y* + 1, −*z* + 1; (ii) −*x* + 1, *y* + 0.5, −*z* + 0.5; (iii) *x* – 1, −*y* + 1.5, *z* – 0.5; (iv) *x*, −*y* + 1.5, *z* – 0.5, (v) *x* + 1, *y*, *z*; and (vi) −*x*, −*y* + 2, −*z*.

**Figure 6:**
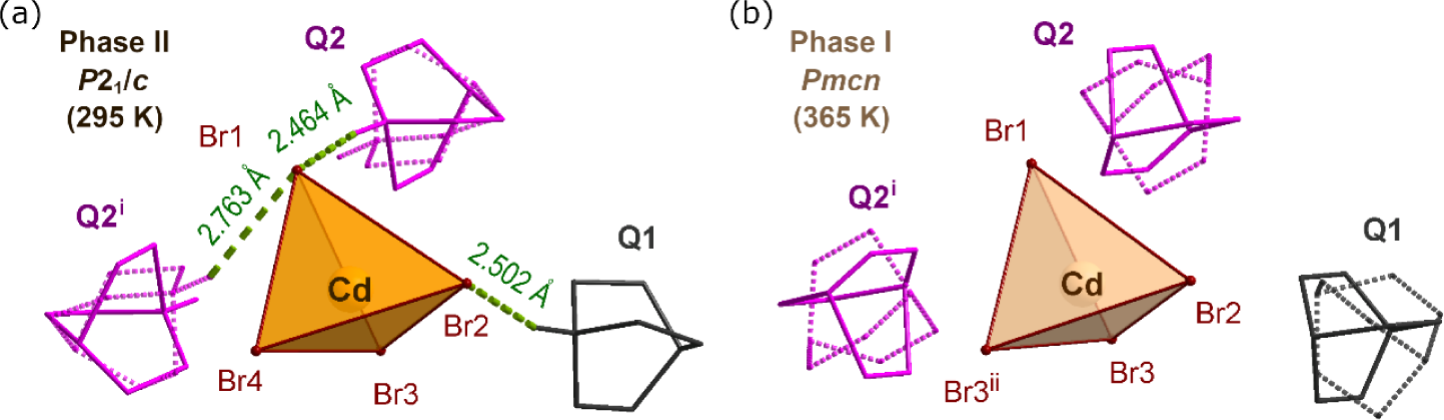
(a) Q_2_CdBr_4_ structure in phase II at 295 K showing the CdBr_4_^2–^ anion with three adjacent Q cations and N—H···Br bonds presented as green dashed lines. (b) Q_2_CdBr_4_ structure in phase I at 365 K. The disordered cations are presented with dotted lines, gray for Q1 and pink for Q2 cations. The symmetry codes: (i) −*x* + 1, −*y* + 1, −*z* + 1; (ii) −*x* + 3/2, *y*, *z*.

The asymmetric unit comprises independent ions: an MBr_4_^2–^ tetrahedron and two protonated quinuclidine cations (Q) (see Figures S1, S4, and S5). As hybrid compounds of the *A*_2_*M*Br_4_-type (*A*–monovalent cation, *M*–metal), they exhibit a typical zero-dimensional (0D) structure of metal halides. Notably, one of the Q cations in the structure is disordered over two positions (marked as Q2 in Figures 4b, 5b and 6a), with an occupancy of 0.5 for each position. The N—H···Br hydrogen bonds (HBs) connect the Q ions with tetrahedra along the *a* and *c* crystallographic directions (Figure 2). These *ac* layers are further linked along the *b* crystallographic direction by C—H···Br HBs. Although all compounds are isostructural at room temperature, their structures undergo different changes during phase transitions (Figures 2, 3, and S3).

### Q_2_CoBr_4_

The step change in unit cell volume around the PT temperature (see Figure S3) confirms that the PT in Q_2_CoBr_4_ is of first-order type. After the reorientation of the cations, the low-temperature ordered structure of phase II is described in space group *P*2_1_/*n*, with a change in the β angle. The modification of crystal axes is as follows: *a*_I_ = *a*_II_, *b*_I_ = *c*_II_, and *c*_I_ = *b*_II_. During the PT, the Q2 cation changes position, creating a new, shorter HB of 2.57 Å (compared to 2.81 and 2.87 Å in phase I) with another Br ion from the cadmium coordinated tetrahedron, N2—H2···Br3. Simultaneously, the Q1 ion also tilts, forming a hydrogen bond with a different CoBr_4_^2–^ ion than in phase I (Figure 4). After these reorientations, N—H···Br bonds along the *a* crystallographic direction are no longer present in the structure (Figure 2a). The selected HB parameters are reported in Table S1. After the PT, the low-temperature ordered phases of Q_2_CoBr_4_ and its previously reported Q_2_CoCl_4_ analogue^15^ become isostructural.

### Q_2_MnBr_4_

The PT of Q_2_MnBr_4_ is of the second-order type, as evidenced by the gradual change in the unit cell volume around the PT temperature (Figure S3). The structure of the low-temperature phase II is described in the space group *P*. The asymmetric unit contains twice as many atoms as in phase I (Figure S5a). Similar to Q_2_CoBr_4_, the structure becomes ordered, but the PT mechanism differs. Half of the Q2 positions are retained, while the other half (Q4) are tilted, and the position of Q1 (Q3) remains unchanged (Figure 5). The Q4 cation is oriented differently than the corresponding Q2 ions in phase I, forming a new N—H···Br bond with a Mn tetrahedron in an adjacent unit cell (Figure 2c). Consequently, N—H···Br bonds are present in both the *a* and *c* crystallographic directions in phase II of Q_2_MnBr_4_.

### Q_2_CdBr_4_

Upon cooling, the behavior of Q_2_CdBr_4_ is distinct from that of the previously described compounds, as it does not undergo a phase transition. However, the disorder of the Q2 ion is frozen in one of the two possible positions (Figure S1a). As a result, unlike in Q_2_CoBr_4_ or Q_2_MnBr_4_, N—H···Br hydrogen bonds are maintained only in the *a* crystallographic direction (Figure 2e).

After the PT to the high-temperature phase I, the structure acquires *Pmcn* space group symmetry. The nonstandard setting of the *Pnma* space group was chosen to preserve the same crystallographic axes as in phase II. At 365 K, both Q cations are disordered over two positions. In phase I, the disordered Q1 ion is also tilted, and additional N—H···Br HBs are likely formed along the *c* crystallographic direction. However, due to the inherent disorder in the structure at this temperature, the H atoms were not included in the structure model (Figures S1c). The mechanism of the PT is similar to that observed in the Q_2_CuCl_4_ structure.^15^ However, a notable difference lies in the direction in which the disordered Q2 ion tilts in phase II (at RT). In Q_2_CuCl_4_, the N—H···Cl bonds are formed with two different Cl ions (Cl2 and Cl3) from the polyhedra, whereas in the Q_2_CdBr_4_ structure, the HBs are created with the same Br ion (Br1) from CdBr_4_^2–^ tetrahedra (Figure 6).

The crystal structures of the Q_2_CoCl_4_^15^ and Q_2_CoBr_4_ systems exhibit distinct behaviors during phase transitions, largely influenced by halide ions. Notably, the phase transition (PT) temperature for Q_2_CoBr_4_ occurs more than 60 K lower than that for Q_2_CoCl_4_, suggesting that the larger bromide ion may stabilize the high-temperature phase, thereby lowering the transition temperature. In Q_2_CoCl_4_, the PT above RT (∼320 K) leads to significant disorder, with protonated quinuclidine cations (Q) exhibiting rotations. Additionally, N—H···Cl bonds at RT are of similar lengths to those at 100 K (3.28–3.38 Å vs 3.23–3.35 Å). Q_2_CoBr_4_ also undergoes a first-order PT but below RT (∼260 K). Phase II of this compound is characterized by an ordered structure, where the reorientation of cations results in shorter hydrogen bonds (N2—H2···Br 3.44 Å vs 3.57–3.79 Å in phase I), indicating a stronger interaction between the cation and the halide anion.

The effect of the presence of halides in the manganese systems is also noteworthy. A similar temperature dependency for the PT is observed in Mn-bromide, where the transition occurs approximately 75 K lower than for the Mn–Cl analogue.^15^ For both structures with manganese atoms, the first-order PT is observed, accompanied by structural ordering. However, Q_2_MnCl_4_ remains disordered in phase II. It is also the only structure among the studied compounds that has three positions for Q ions in its lower-temperature phase II. For Q_2_MnBr_4_, the PT induces ordering with more pronounced structural changes, as one of the Q cations tilts significantly to form new N—H···Br bonds with an adjacent Mn tetrahedron. This difference suggests that the bromide anion may facilitate more dynamic reorientations and stronger bonding compared to chloride. In addition, in all structures where the PT temperature is above RT, the structural behavior pattern is analogous, regardless of the halide ion present in the compound.

The observation described above applies to the compounds Q_2_CoCl_4_, Q_2_CuCl_4_, and Q_2_CdBr_4_. During the PT, their structure changes from orthorhombic (high-temperature phase I) to monoclinic (phase II). In phase II, at RT, they are characterized by Q ion disorder (tilting or rotation), which disappears as the temperature decreases to 100 K. Overall, the comparison between the Cl and Br systems highlights how the halide size and bonding strength influence both the thermal stability and the nature of the phase transitions in these Q_2_*M*Cl_4_ and Q_2_*M*Br_4_ compounds. These structural variations underline the role of the halide ion indictating the behavior of coordination compounds, particularly in terms of temperature-induced disorder and reorientation dynamics.

### IR and Raman Spectroscopy

**Figure 7:**
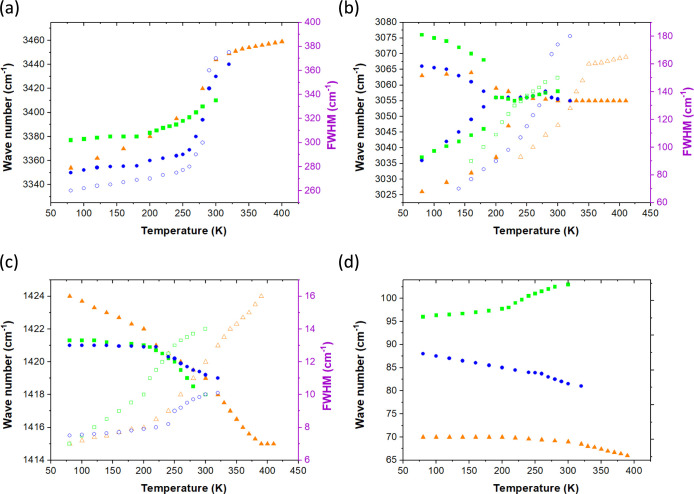
Wavenumber (full figures) and their corresponding fwhm changes (empty figures) for (a) NH stretching, (b) CH_2_ stretching, (c) NH and CH_2_ deformation modes, (d) lattice modes in Q_2_CoBr_4_, (full and empty blue dots), Q_2_MnBr_4_ (full and empty green squares), and Q_2_CdBr_4_ (full and empty orange triangles).

We present a set of spectroscopic (IR and Raman) studies of the quinuclidinium metal bromide family. The IR and Raman spectra of the samples at RT are presented in Figures S6 and S7, respectively. These are followed by temperature-dependent IR spectra in Figures S8–S10 for Q_2_CoBr_4_, Q_2_MnBr_4_, and Q_2_CdBr_4_, respectively, while the analogous Raman spectra are shown in Figures S11–S13. Table S4 collects the observed IR and Raman wavenumbers along with their assignments. The symbols used in the text are explained in the footnote of this table. The assignments are based on the data available for compounds containing Q and similar cations (e.g., dabco) and metal halides, specifically referring to the family of Q*M*Cl (quinuclidinium-metal chlorides).^13−15,22−25^ The observed modes originate from internal vibrations of the organic cations, metal-bromide tetrahedra, and lattice modes. Temperature-induced changes for selected modes are presented in Figure 7a–c.

Further, we have focused on the specific features of the spectra and the influence of temperature, specifically during PT. The influence of the PT on both IR and Raman spectra is observed in the temperature range indicated by the DSC measurements during heating.

The comparison of the bands’ intensities in IR vs Raman spectra confirms the centrosymmetric arrangement, as in IR the asymmetric modes dominate, whereas in Raman they are of little intensity.

As evidenced by Figures S8–S10 and 7a, the hydrogen bonding (HB) is medium-strong in all three crystals. They are a bit shorter (red-shifted by about 30–50 cm^–1^) than the HBs found in the analogous Q*M*Cl family.^14,15^ They are of comparable length, as their stretching modes have a center of gravity located in every crystal within 3300–3500 cm^–1^ and also around 3060–3050 cm^–1^, being almost the same in Q_2_CoBr_4_ and Q_2_CdBr_4_, and a bit longer in Q_2_MnBr_4_, which is as expected since the Mn-based compound, with the smallest Mn ion in the series, has the most compact structure. These two main bands correspond to two HB groups with varying distances: ca. 3.6 and 3.3–3.4 Å, respectively.

The intensity of HB-related bands within 3300–3500 cm^–1^ is the highest in Q_2_CoBr_4_ sample because this material has the narrowest distribution of HBs compared to the Mn- and Cd-analogue, which confirms the X-ray crystal determination data. Additionally, the shorter HBs in all three materials are superimposed with C–H stretching vibrations, having maxima at around 3060 cm^–1^.

The vibrations of Q cations in all three materials are found at very comparable wavenumbers and show a very similar structure, which means that Q symmetry is alike despite differences in point groups and that their bond lengths are basically preserved regardless of the inorganic net. The temperature-dependent IR and Raman measurements prove that Q cations are already disordered at the lowest measured temperature, for example, the fwhm (full width at half-maximum) of NH stretching modes is as high as 260 cm^–1^ in Q_2_CoBr_4_ (Figure 7a). This is in contrast to the QMCl series, wherein Q_2_CoCl_4_ and Q_2_CuCl_4_ became ordered at 80 K, but it is similar to Q_4_Pb_3_Cl_10_.^15^

The PT is well manifested in the spectra regarding HBs in the form of N—H···Br. Figure 7a presents the wavenumber changes of stretching modes for longer HBs upon a temperature increase across all three crystals. The smallest shift belongs to the Q_2_MnBr_4_ crystal (ca. 30 cm^–1^), which stems from the fact that the Q cations have more space compared to other analogues and do not need to adjust that much to fit into a new structure. The shift with temperature reaches merely 30 cm^–1^ in Q_2_MnBr_4_; however, it goes up to around 100 cm^–1^ in Q_2_CoBr_4_ and Q_2_CdBr_4_, whose structures are more compact and comparable. However, it is still surprising to see the fwhm change of HBs in Q_2_CoBr_4_ which increases from 260 to 380 cm^–1^, resulting in a span of 120 cm^–1^. We have already been amazed by the significant value of 150 cm^–1^ found in Q_2_CuCl_4_.^15^ Similarly to the cited Cu-based material, the crystal of Q_2_CoBr_4_ is found to possess almost freely and undisturbedly rotating Q cations in phase I, which get quite well ordered at LT, as evidenced by the splitting of the C–H bands, as described below. It is hard to estimate the fwhm changes for the other two compounds, since they are mostly superimposed with C–H bands or are of little intensity. The PT changes are best pronounced for Q_2_CoBr_4_ as expected from the DSC results, quite faint but still noticeable for Q_2_MnBr_4_ and for Q_2_CdBr_4_ the changes occur ahead of the PT, indicating that the PT does not depend only on the rearrangement of HBs. The changes of NH stretch in the Cd-based sample are constant from the lowest temperature to the highest point, with a clear ongoing process of dynamic reorientation. The PT is also well visualized in the region of NH deformation vibrations combined with C–H modes, as presented in Figure 7c.

'`'In general, all three compounds show a splitting of the C–H bands associated with CH and skeleton vibrations of the Q cation. As presented in Figure 7b, the splitting reaches 20 cm^–1^ for Q_2_CoBr_4_, 40 cm^–1^ for Q_2_MnBr_4_, and 35 cm^–1^ for Q_2_CdBr_4_; however, above PT, at higher *T*, they all converge to more or less the same wavenumber. The splitting is repeated for other C–H-related modes at lower wavenumbers in all three materials in both IR and Raman spectra. This is proof of the ordering of the Q cations at LTs and their disordering with the temperature increase. However, the merging of the 3060 cm^–1^ band into one happens before the PT in Co- and Cd-based crystals. The PT is present there as a small jump in the wavenumber position. This is in agreement with the results obtained from the HBs region: the Q cations in Q_2_CoBr_4_ are similar to each other and better ordered at LT than in the other two samples, and the PT in Q_2_CoBr_4_ is inherent to Qs, while in the latter two, the PT mechanisms are more complex. The splitting values cited above for Q_2_CoBr_4_ are comparable again with those for Q_2_CuCl_4_, but they are much stronger than those for Mn- and Cd-based crystals, which means that the corresponding Q cations differ much more from each other, probably trying to fit into the newly formed structures. It is also worth noting the extreme broadening of the bands with *T* increase, particularly huge for Q_2_CoBr_4_ (ca. 120 cm^–1^), which has never been observed before in such systems.

The bands observed at ca. 1420 cm^–1^ originate from NH and CH_2_ deformation vibrations (see Figure 7c). Unlike shown before and thus unexpectedly, the biggest changes occur in Q_2_CdBr_4_, reaching 10 cm^–1^ in wavenumber shift, followed by 3 cm^–1^ in Q_2_MnBr_4_ and 2 cm^–1^ in Q_2_CoBr_4_ and with fwhm changes following the same order: 10, 8, and 4 cm^–1^, respectively, being comparable to the Q*M*Cl series. It is an indication of the fact that the internal bonds in the Q cations of the Cd crystal are most perturbed by the structural changes due to the shortest (and thus strongest) HBs in the series, which are being dragged and pulled sideways by those HBs.

The changes observed in the region of lattice vibrations are pictured by referring to one band in Figure 7d based on Raman spectra. All the bands experience a wavenumber shift due to the symmetry change as well as become broader as a result of the disorder, but at the same time, the bands below 100 cm^–1^ gain much intensity. This is clearly the result of the change in crystal symmetry. The band present within 70–100 cm^–1^ originates from *M*–Br deformations and Q librations. It undergoes a wavenumber shift by 5 cm^–1^ in Cd-based crystals and by 10 cm^–1^ in Co- and Mn-based crystals; however, for Mn-based crystals, the shift is toward higher wavenumbers, unlike in Co and Cd samples. This means the *M*–Br distances get shorter with the temperature increase, which results from weaker interactions with HBs. Indeed, the HBs do get longer in Q_2_MnBr_4_ compared to Q_2_CoBr_4_ and Q_2_CdBr_4_, as imposed by the X-ray data. This shift value for the Cd compound is a rather expected result, as Cd is the heaviest of the three metal ions. The PT is present slightly ahead of the temperatures indicated by DSC in Q_2_CoBr_4_ and Q_2_CdBr_4_, while it is at the PT temperature for Q_2_MnBr_4_. This may suggest that the impact of the inorganic network on the PT is strongest in the latter compound, which is in agreement with the weakening of the interactions between the manganese-bromide tetrahedra and HBs.

### Dielectric Measurements

**Figure 8:**
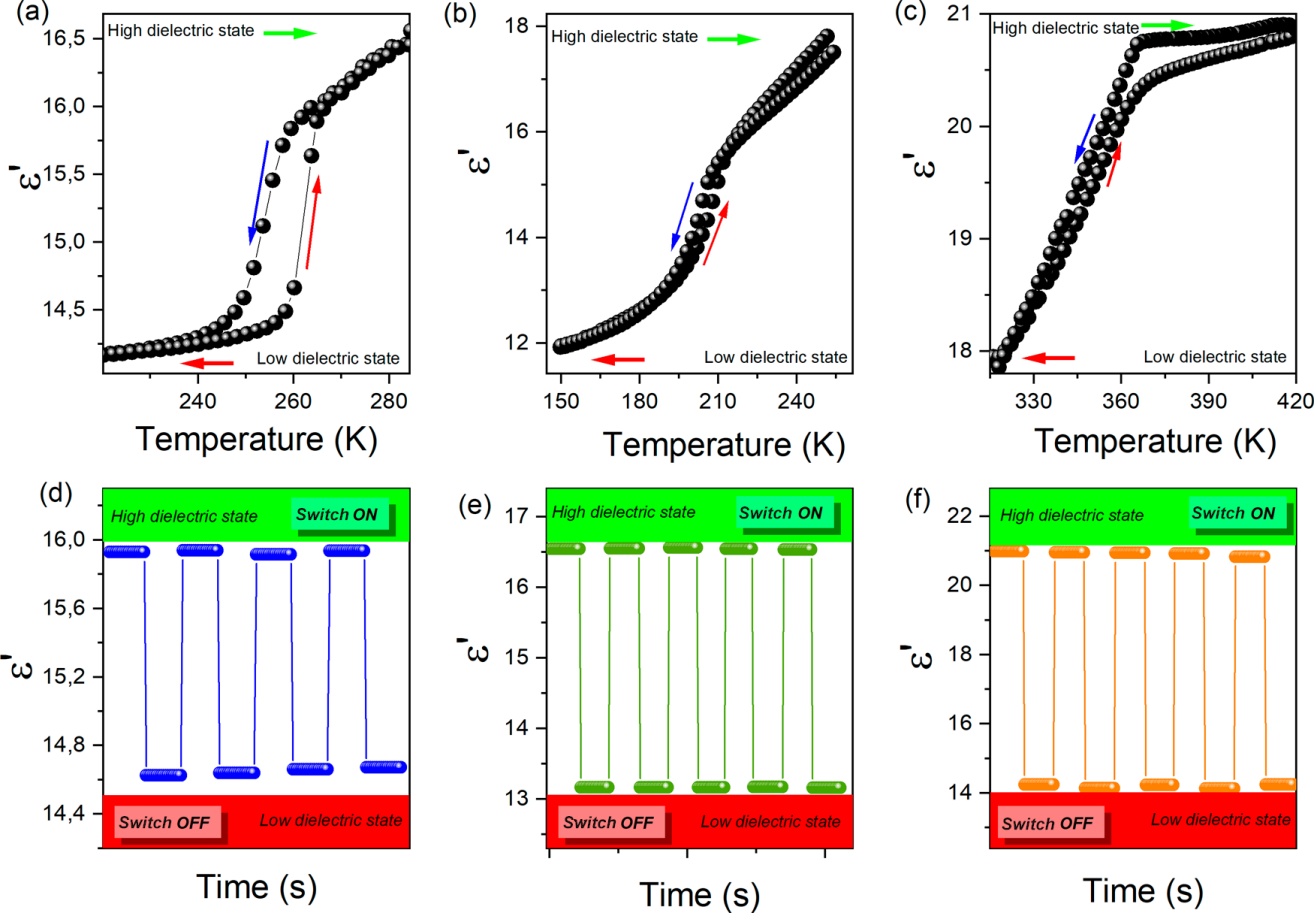
Respective temperature dependences of the real part of dielectric permittivity (ε’) in the *T*-range of the order–disorder phase transition of Q_2_CoBr_4_ (a), Q_2_MnBr_4_ (b), and Q_2_CdBr_4_ (c). The repeatability cycles of the thermal switching of ε’ depicted by its temperature variable values for Q_2_CoBr_4_ (d), Q_2_MnBr_4_ (e), Q_2_CdBr_4_ (f) measured at 1 MHz heating/cooling cycle.

The dielectric measurements of the complex dielectric permittivity were performed on powder-pressed Q_2_CoBr_4_, Q_2_MnBr_4_, and Q_2_CdBr_4_ samples. In the heating/cooling mode, the temperature-dependent dielectric response reveals a pronounced step shape near the phase transition temperature during heating and cooling. The real part of the dielectric constant increases sharply at 261 K (Q_2_CoBr_4_), 205 K (Q_2_MnBr_4_), and 350 K (Q_2_CdBr_4_) upon heating, and decreases at 253 K (Q_2_CoBr_4_), 201 K (Q_2_MnBr_4_), and 345 K (Q_2_CdBr_4_) (Figure 8). Subsequently, for Q_2_CoBr_4_, the permittivity exhibits thermal hysteresis (∼10 K), strongly confirming the occurrence of a first-order phase transition, which is consistent with thermal analyses. The temperature trends for Q_2_MnBr_4_ and Q_2_CdBr_4_ exhibit similar patterns but without well-defined thermal hysteresis, suggesting second-order phase transitions.

According to the XRD studies, the thermally activated motions of Q cations likely trigger changes in the related dipole moments, resulting in a switchable dielectric response. This thermal switching of the dielectric constant can be highly repeatable (Figure 8d–f). The scale of switching the *ε′* values is comparable in all compounds. What is more, the dielectric switching in Q_2_MBr_4_ compounds is completely reversible between high and low dielectric states. After several ON/OFF cycles, the dielectric constant *ε*′ demonstrates significant reversible dielectric switching and fatigue resistance. Importantly, the ability of these compounds to switch between high and low dielectric states makes them excellent candidates for molecular electrically switchable materials.

The relaxation mechanism of Q_2_*M*Br_4_ compounds was analyzed by the electric modulus spectra. The complex electric modulus is defined as M*(ω), which is the reciprocal of the complex dielectric permittivity, ε*(ω):
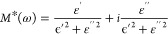
1where *M*′(ω) and *M*″(ω) are the real and imaginary parts of the electric modulus, respectively.

Figures S14 in the ESI shows the variation of *M*″(ω) for Q_2_*M*Br_4_ compounds as a function of frequency over a wide temperature range. The *M*″(ω) spectra exhibit distinct peaks at all investigated temperatures, indicating the relaxation frequency of the materials. The value of *M*″(ω) increases with frequency, reaching the relaxation frequency. When the temperature increases, the peaks shift toward higher frequencies, suggesting that the relaxation behavior of Q_2_*M*Br_4_ compounds is thermally activated. To elucidate the nature of the relaxation behavior, the *M*″(ω) data were analyzed, and the activation energies were calculated. The plot of relaxation frequency *f*_max_, as a function of the reciprocal of temperature follows Arrhenius behavior (insert in S14 ESI). The activation energies calculated from the linear fit (see Figure S14) are *E*_a_ ∼ 0.95(±0.05) eV, *E*_a_ ∼ 0.68(±0.05) eV, and *E*_a_ ∼ 0.48(±0.05) eV for Q_2_CoBr_4_, Q_2_MnBr_4_, and Q_2_CdBr_4_, respectively. The activation energy in the disordered phase for Q_2_CoBr_4_ is comparable to that of Q_2_CoCl_4_,^15^ suggesting that the observed processes in both compounds are similar and are related to the ordering of Q molecules in the low-temperature phase.

Figures S15–S17 show the frequency-dependent real part of the dielectric constant (*ε*’) and dielectric loss (*tanδ*) over a wide temperature range. It is observed that in the low-frequency region, the value of *ε*’ is higher and decreases significantly at higher frequencies. Dielectric permittivity depends on various types of polarization, including ionic, orientational, interfacial, and electronic polarization. The interfacial polarizations dominate *ε*’ in the low-frequency region and account for the lower values of *ε*′(*ω*), while the electronic and ionic polarizations contribute more in the high-frequency domain. For Q_2_*M*Br_4_ samples, at a particular frequency, the values of *ε*′(*ω*) increase with temperature, indicating the presence of thermally activated charge carriers. In the low-temperature region, *ε*’(*ω*) increases as the temperature rises, while at high temperatures, *ε*’ becomes almost temperature independent.

The loss tangent shows similar behavior for both Q_2_CoBr_4_ and Q_2_CdBr_4_ compounds, with a relaxation peak observed in the spectra of *tanδ*. At low frequencies, the loss tangent increases with frequency, reaching a maximum value before decreasing as the frequency continues to rise. Generally, the maximum in the loss tangent occurs when the hopping frequency coincides with the frequency of the applied external field. Due to the dominant dipolar polarization at low temperatures, loss tangent peaks appear in the low-frequency region, shifting toward higher frequencies with increasing temperature, indicating that dipolar polarization is a thermally activated process. For Q_2_MnBr_4_, no relaxation peaks were observed in the loss tangent spectra.

The dielectric relaxation can be described by the Arrhenius equation: *f*_max_ = *f*_0_ exp(*E*_a_/*k*_B_*T*), where *E*_a_ is the activation energy required for the relaxation process and *f*_0_ is the pre-exponential factor. As shown in Figure S18, the fitted dielectric relaxation energies are *E*_a1_ = 0.274 and *E*_a2_ = 0.467 eV for Q_2_CoBr_4_, and *E*_a1_ = 0.263 eV and *E*_a2_ = 0.330 eV for Q_2_CdBr_4_. The differing activation energies derived from the modulus representation and the dielectric loss tangent formalism suggest distinct characteristics of the observed processes. According to the XRD analysis, phase I of the Q_2_MBr_4_ compounds is characterized by the presence of at least one quinuclidine (Q) cation that is disordered over two energetically equivalent positions. The activation energies obtained from the modulus representation can be attributed to the energy required for the Q cations to relocate within phase II at lower temperatures. Additionally, in Q_2_CoBr_4_ and Q_2_CdBr_4_, as the temperature decreases, all ions exhibit a tilting motion (see Figure 2a,b,e,f). It can be deduced that the relaxation processes and the corresponding activation energies observed in the spectra of tanδ may be associated with the rotations of the Q cations.

### ^1^H Spin–Lattice Relaxation Studies

**Figure 9:**
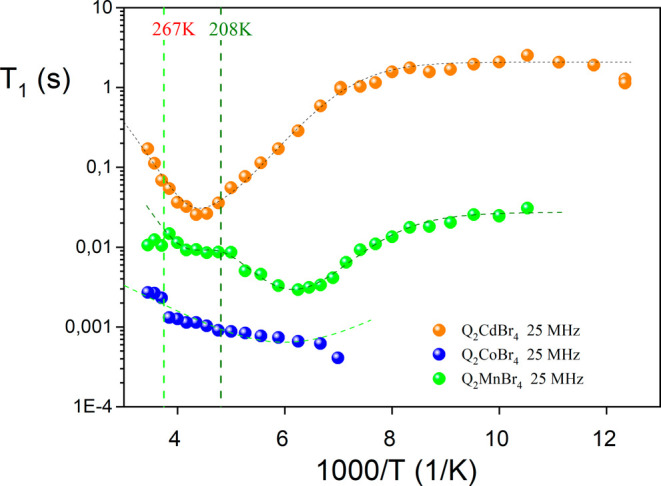
Temperature dependence of the proton spin–lattice relaxation time of Q_2_*M*Br_4_ compounds measured at 25 MHz. In ow temperatures, the deflexion is present due to the dominant quadrupolar mechanism. Two corresponding phase transitions are marked.

The temperature dependencies of the ^1^H NMR spin–lattice relaxation time, *T*_1_ for the studied bromide compounds Q_2_MBr_4_ are shown in Figure 9. The measured one-exponential magnetization recovery functions were found for all compounds. For Q_2_CoBr_4_, the measured *T*_1_ values are very short and approach the limit of measurement capabilities of the used spectrometer, likely due to the dominance of strong paramagnetic interactions in the samples. Below 140 K, no signal of FID (Free Induction Decay) is detected. Despite this limit uncertainty of the experiment, we have applied an analysis approach previously used for paramagnetic samples.^26^ In the case of a predominant paramagnetic interaction, the proton–proton dipole–dipole interaction may be neglected. For the final fitting procedure, we applied a simplified expression for the ^1^H spin–lattice relaxation time^24^
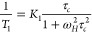
2and the obtained dynamic parameters from the fitting procedure are as follows: for Q_2_CoBr_4_: *E*_a,1_ = 6.46 kJ/mol, *K*_1_ = 4.88 × 10^11^ Hz^2^, as shown in Figure 9 by the dashed blue curve.

In turn, the Q_2_MnBr_4_ and Q_2_CdBr_4_ bromide compounds have longer longitudinal relaxation times than those previously described. Upon heating from low temperatures, a slight shortening in *T*_1_ relaxation times can be observed, and before the phase transition, the measured data points form the relaxation time minimum. The analysis of the *T*_1_ relaxation time dependence within the temperature range of 84 and 290 K is conducted under the assumption that the overall relaxation consists of two relaxation mechanisms: one attributed to classical ^1^H dipole–dipole interactions and the other due to the interaction of hydrogen nuclei with quadrupole bromide nuclei. The characteristic smoothing observed at low temperatures is assigned to that second relaxation mechanism, while the onset of the relaxation minimum before the phase transition results from the dominance of the classical relaxation mechanism at higher temperatures. In the case of the cadmium bromide compound, a clear asymmetry is evident, with a smaller slope on the right side of the minimum in longitudinal relaxation times at lower temperatures. This asymmetry is likely caused by the presence of two different types of classical interactions resulting from dynamically unequal quinuclidine cations in a 1:1 ratio. Therefore, to fit the theoretical curve, it is necessary to consider two classical relaxation mechanisms of the dipole–dipole interaction of two different nonequivalent quinuclidine cations.

In the case of the Q_2_MnBr_4_ compound, instead of a single asymmetric minimum in the longitudinal relaxation times, two well-separated minima of longitudinal relaxation times are present. In contrast to the data above, the difference in the depth of these two minima suggests that the relation between the dynamically unequal quinuclidine cations is closer to a ratio of 3:1. Similar to the case of the cadmium compound, fitting the theoretical curve requires considering two classical relaxation mechanisms of the dipole–dipole interaction.

The quadrupole relaxation for the Q_2_MnBr_4_ and Q_2_CdBr_4_ samples was assumed to be temperature-independent. The effective modulation of the proton-bromine nucleus dipole–dipole coupling contributes to the nearly constant ^1^H NMR relaxation times, which is reflected in the smoothing observed in Figure 9, despite the very long correlation time *τ*_c_ at low temperatures.

To analyze the entire temperature dependence of the measured relaxation times, we use an equation where two components are related to the two different classical dipole interactions, and the third one is related to the quadrupole interaction observed at the lowest temperatures. The fitting procedure for the entire analyzed temperature dependence of the *T*_1_ relaxation time is performed using the sum of these three components:
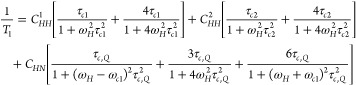
3where *ω*_Cl_ is the bromine nuclei Larmor frequency. The correlation time *τ*_c,Q_ is defined as *τ*_c,Q_^–1^_*=*_*τ*_c_^–1^ + *R*_Q_, where *R*_Q_ represents the quadrupolar spin–lattice rates of the bromine nuclei. After the numerical fitting to the experimental data (black dashed line and olive dashed line, respectively, in Figure 9), the following parameters are found for the cadmium compound: *E*_a1_ = 16.98 kJ/mol, τ_c1_ = 3.28 × 10^–13^ s, the = 2.07 × 10^9^ s^–2^, *E*_a2_ = 25.4 kJ/mol, τ_c2_ = 1.25 × 10^–14^ s, the = 2.07 × 10^9^ s^–2^, C_HQ_ = 2.67 × 10^7^ s^–2^. For the manganese compound, the parameters are *E*_a1_ = 13.67 kJ/mol, τ_c1_ = 1.638 × 10^–13^ s, the = 3.16 × 10^10^ s^–2^, *E*_a2_ = 21.13 kJ/mol, τ_c2_ = 9.0 × 10^–14^ s, the = 8.26 × 10^9^ s^–2^, C_HQ_ = 2.5 × 10^9^ s^–2^ with applied *R*_Q_^–1^ = 6.749 × 10^–7^ s. The effective modulations of the proton-bromide nuclei dipole–dipole coupling contribute to the near-constancy of the ^1^H relaxation times (as shown by the deflexion in Figure 9), despite the very long correlation time *τ*_c_ at low temperatures. A notable analogy can be seen in the obtained values of the dipole–dipole interaction dynamics parameters for both studied bromine compounds, with a significant difference being the much smaller parameter of the quadrupole relaxation constant in the manganese compound. In the case of the manganese compound, the paramagnetic properties begin to prevail over the dominance of the quadrupole interaction at low temperatures. These obtained dynamical parameters are similar to those of compounds with present quadrupole interaction.^27,28^ Finally, let us note that only for the cobalt compound can a sudden change in the dependence of relaxation times on temperature be seen in the phase transition of the first kind, indicating a sudden change in the dynamics of the cation as well. In the case of the manganese compound, the presence of the phase transition of the second kind has a negligible effect on the dynamics of the cation, indicating perhaps only changes in the dynamics of the anion network.

### *M*_2_ of the ^1^H NMR Line

**Figure 10:**
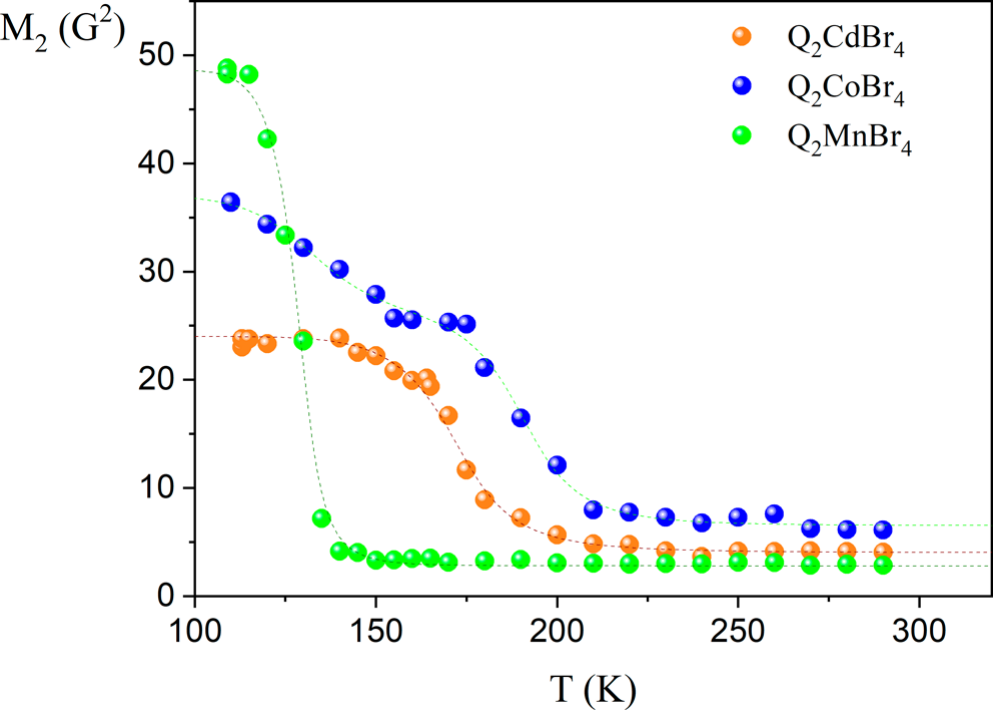
Moment of the ^1^H NMR lines for Q_2_*M*Br_4_ compounds.

The temperature dependences of the second moment *M*_2_ of the ^1^H NMR line are presented in Figure 10. For all studied samples, similar reductions of the ^1^H NMR line occur around 200 K for Q_2_CoBr_4_, around 175 K for Q_2_MnBr_4_, and around 145 K for Q_2_CdBr_4_. At lower temperatures, a further reduction is observed for the paramagnetic Q_2_CoBr_4_, resembling the reduction observed at low temperatures for its chloride analog.^15^ Possible phase transitions do not significantly affect the observed reductions; for example, in the case of Q_4_Pb_3_Cl_10_,^15^ the phase transition at 185 K does not affect the observed temperature dependence of the experimental points, which may indicate a relationship between this phase transition and the dynamic changes of the anions.

In all studied compounds, a significant reduction from 24 × 10^–8^ T^2^ to approximately 6.2 × 10^–8^ T^2^ is observed, reaching near room temperature. Notably, high values were determined for the paramagnetic Q_2_CoBr_4_ sample, with a considerable reduction likely caused by the possible dipole–dipole interaction of protons with unpaired electrons. It is important to note that the calculations of the second moment for the rigid state of quinuclidine cation’s molecular structures, based on structural crystallographic data, were made previously in a paper with the chloride analogy compounds^15^ and the assumed distances between proton and carbon or nitrogen positions (C—H = 1.09 Å and N—H = 1.04 Å). The theoretical second moment value for the only diamagnetic rigid lattice for the quinuclidine cation was calculated to be *M*_2_^Rigid^ = 18.06 × 10^–8^ T^2^ (considering only *intra* value without *inter* contributions). This clearly indicates that the similar reductions in the second moment ^1^H NMR line widths for all three compounds reflect the onset of faster molecular reorientation of the frozen cations. The different temperatures at which these *M*_2_ reductions occur suggest that Q_2_CdBr_4_ first starts to move, while Q_2_CoBr_4_ exhibits the slowest response. The observed three reductions of the ^1^H NMR line width correspond well to the theoretical *M*_2_ value for a rigid structure, particularly in the case of Q_2_CoBr_4_, where the influence of additional interactions between protons and unpaired electrons can be observed. For calculating the dynamic parameters, the found reductions of the second moment may be analyzed as a temperature dependence based on the formula:
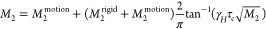
4where the correlation time fulfills the Arrhenius law *τ*_*c*_*= τ*_*o*_ exp(*E*_*a*_/*RT*), *M*_2_^rigid^ and *M*_2_^motion^ are the second moment values before (rigid) and after (motion) the onset of a given motion, respectively. From the fitting, the following parameters are obtained: for the cadmium compound: *E*_*a*_ = 17.92 kJ/mol and τ_c0_ = 3.71 × 10^–11^ s; for the manganese compound: *E*_*a*_ = 21.21 kJ/mol and τ_c0_ = 1.74 × 10^–14^ s. In the case of Q_2_CoBr_4_, it was possible to determine dynamic parameters for two reductions: the first, *E*_*a*1_ = 10.8 kJ/mol and τ_c10_ = 4.18 × 10^–10^ s, and the second, *E*_a2_ = 25.04 kJ/mol and τ_c20_ = 1.31 × 10^–12^ s (see fitting curve in Figure 10). For the cadmium compound, fitting with only one reduction formula was used, although one can try to see the overlap of two reductions related to the cation nonequivalence found in the *T*_1_ time measurements. A very rapid, sharp reduction for the manganese compound occurred at the lowest temperatures of the three compounds, observed with a significant second moment value more than twice that determined from the rigid crystal structure, indicating an effective relaxation path via unpaired electrons. All the above-described temperature dependencies of the reduction of the second moment *M*_2_ confirm the possibility of starting some axial movement of quinuclidine cations. Assuming that at high temperatures usually *M*_2_^inter^ ∼ 1 × 10^–8^ T^2^, the quinuclidine cation reorientation in the case of Q_2_CdBr_4_ is closest to tumbling, while the most axial movement occurs for Q_2_CoBr_4_. It is visible that the presence of phase transitions occurs just within the range of axial motions of the cations, and their influence on them is negligible.

### EPR study of Q_**2**_**MnBr**_**4**_

The EPR fine structure of the high-spin Mn^2+^ ion can be generally described by the following spin Hamiltonian: , where the first term, with the spectroscopic coefficient *g* (*g-*facto*r*), determines the Zeeman effect, while the axial (*D*) and rhombic (*E*) parameters characterize the splitting of energy levels in the zero magnetic field *B*. Since the parameters of the spin Hamiltonian are highly sensitive to any changes in the distance and symmetry of the coordination environment of the paramagnetic ion, the EPR spectrum of Mn^2+^ serves as a valuable tool for detecting phase transitions.^15,29^Figure S19 (ESI) presents EPR Mn^2+^ ion spectra of powdered samples across a selected temperature range. These spectra are very complex and composed of many overlapping broad lines, as expected for MnBr_4_ complexes.^30,31^ Moreover, no clear changes are visible in the EPR spectra at the phase transition temperature.

**Figure 11:**
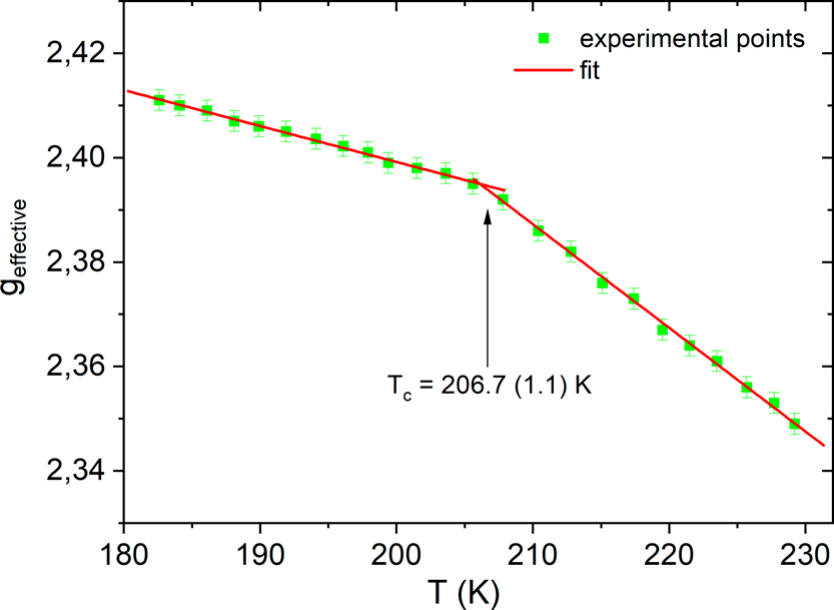
Temperature changes of the *g*_effective_ parameter for Q_2_MnBr_4_.

To analyze the spectra more precisely, we have introduced the parameter *g*_effective_ (dependent on parameters *g*, *E,* and *D*), which defines the spectroscopic splitting coefficient at the local EPR microwave absorption maximum (d*I*/d*B* = 0), as presented in Figure S19 (ESI). The behavior of the *g*_effective_ parameter vs temperature, shown in Figure 11, clearly indicates a change in the slope of *g*_effective_(*T*), which is related to the phase transition at 206.7 ± 1.1 K. The absence of temperature hysteresis and the continuous change in the *g*_effective_ value at the phase transition temperature clearly confirm that this is a second-order phase transition. Moreover, the shift of *g*_effective_ vs temperature indicates alterations in the distances/angles between the ions forming the MnBr_4_ complexes, which is consistent with X-ray diffraction studies.

The integral intensity *I* of the EPR spectrum for localized paramagnetic complexes should be proportional to the Curie law, given as *I*(*T*) ∝ *C*/*T*, where the Curie constant *C* is dependent on the *g*-factor.

The change in the slope of the I(1/*T*) function at about 206.7 K, presented in Figure S20 (ESI), also indicates a phase transition of the second order and the additional distortion for MnBr_4_ complexes that occurs at the phase transition.

## Conclusions

The investigation of the structure and thermal, electric, and magnetic properties of the organic–inorganic (C_7_H_14_N)_2_MBr_4_ (*M* = Co, Mn, Cd) crystals has been performed. The crystal structure analyses of compounds Q_2_CoBr_4_, Q_2_MnBr_4_, and Q_2_CdBr_4_ reveal distinct crystal symmetry changes during phase transitions. All three compounds crystallize in the centrosymmetric space group *P*2_1_/*c* at room temperature, exhibiting a 0D metal halide structural framework. Q_2_CoBr_4_ undergoes a first-order phase transition, as evidenced by a step change in unit cell volume, resulting in a new low-temperature ordered structure with the *P*2_1_/*n* space group. In contrast, Q_2_MnBr_4_ undergoes a second-order phase transition, with gradual changes in the unit cell volume, acquiring the low-symmetry *P* space group and having twice as many atoms in the asymmetric unit. Q_2_CdBr_4_, contrary to the others, undergoes a phase transition above room temperature, which leads to an increase in the disorder among Q ions, resulting in the symmetrically higher *Pmcn* space group. The phase transitions in the Q_2_MBr_4_ compounds involve distinct reorientation of Q ions and reorganization of hydrogen bonds for each compound, highlighting the diversity of phase transition mechanisms in these hybrid compounds. These findings underscore the complexity of structural dynamics in organic–inorganic hybrids and suggest potential for tuning their properties for various applications

However, the mechanisms behind these transformations differ due to the distinct behavior of quinuclidine in the low-temperature phases. Furthermore, EPR measurements have confirmed the second-order phase transition in the Q_2_MnBr_4_ compound and the distortion of MnBr_4_ complexes near the phase transition region. Notably, the thermal and dielectric stabilities of the crystals revealed switch-ON/OFF responses between low and high dielectric states, suggesting their potential applications as switchable and sensing materials.

## Supporting Information Available

Additional structural details for complexes Q_2_MBr_4_, dielectric permittivity, and electrical modulus analysis; Raman spectra and EPR spectra for Q_2_MBr_4_. (PDF)
