# Supplementary material for: Structural Diversity and Stability of Organic–Inorganic Hybrid Quinuclidine-Based Metal Bromides
Source: Inorg Chem. 2025 Apr 11;64(15):7422–36. doi: 10.1021/acs.inorgchem.5c00039 (PMC12015820; doi:10.1021/acs.inorgchem.5c00039)
Supplement: Supplementary file 1 — ic5c00039_si_001.pdf [file ic5c00039_si_001.pdf]

Supporting Information

Structural Diversity and Stability of Organic-Inorganic Hybrid Quinuclidine-based Metal Bromides

Ewelina Jach[1], Dorota A. Kowalska[2], Monika Trzebiatowska[2], Wojciech Medycki[3], Adam Ostrowski[3], Waldemar Bednarski[3], Marek A. Gusowski[1], Piotr Staniorowski[4], Adam Bartosiewicz[1], Urszula Dieu[1] and Agnieszka Ciżman[1*]*

[1] Department of Experimental Physics, Wrocław University of Science and Technology, 27 Wybrzeże Wyspiańskiego, Wrocław 50-370, Poland

[2] Institute of Low Temperature and Structure Research, Polish Academy of Sciences, Okólna 2, 50-422 Wrocław, Poland

[3] Institute of Molecular Physics, Polish Academy of Science, M. Smoluchowskiego 17, 60-179 Poznań, Poland

[4] Institute of Experimental Physics, University of Wrocław, Pl. M. Borna 9, 50204 Wrocław, Poland

email:agnieszka.cizman@pwr.edu.pl
email:ewelina.jach@pwr.edu.pl

Figure content:

Figure S1. The asymmetric unit of the Q_2CdBr_4 with atom numbering scheme (a) in phase II at 100 K with frozen motion of Q2 moiety, (b) in phase II at 295 K with disorder of Q2 and (c) in phase I at 365 K with disordered Q1 and Q2 ions (presented in transparent color and with dashed bonds). The symmetry code: (i) $-x+1/2$, y, z. The N—H···Br bonds are presented as green dashed lines.

Figure S2. The powder X-ray diffraction data of studied compounds.

Figure S3. The volume changes around PT for Q_2CoBr_4, Q_2MnBr_4 and Q_2CdBr_4 derived from X-ray diffraction measurements.

Figure. S4. The asymmetric unit of the Q_2CoBr_4 with atom numbering scheme: (a) ordered phase II at 100 K and (b) phase II at 295 K with disordered Q2 cation (presented in transparent color and with dashed bonds). The N—H···Br bonds are presented as green dashed lines.

Figure S5. The asymmetric unit of the Q_2MnBr_4 with atom numbering scheme: (a) ordered phase II at 100 K and (b) phase II at 295 K with disordered Q2 cation (presented in transparent color and with dashed bonds). The N—H···Br bonds are presented as green dashed lines.

Figure S6. The RT IR spectra of Q_2MBr_4 crystals.

Figure 7. The RT Raman spectra of Q_2MBr_4.

Figure S8. The temperature-dependent IR spectra of Q_2CoBr_4.

Figure S9. The temperature-dependent IR spectra of Q_2MnBr_4.

Figure S10. The temperature-dependent IR spectra of Q_2CdBr_4.

Figure S11. The temperature-dependent Raman spectra of Q_2CoBr_4.

Figure S12. The temperature-dependent Raman spectra of Q_2MnBr_4.

Figure S13. The temperature-dependent Raman spectra of Q_2CdBr_4.

Figure S14. Variation of imaginary part of the complex electric modulus[M ′′(ω)] with frequency (f) for (a) Q_2CoBr_4, (b) Q_2MnBr_4 and (c) Q_2CdBr_4. Insert: ln(f_{max}) versus 1000/T plot obtained from the complex electrical modulus plot showing Arrhenius behavior.

Figure S16. Frequency-dependent real part of (a) dielectric constant (ε′) and (b)loss tangent (tanδ) at over the temperature range 300–200 K for Q_2MnBr_4 compound.

Figure S17. Frequency-dependent real part of (a) dielectric constant (ε′) and (b)loss tangent (tanδ) at over the temperature range 300–200 K for Q_2CdBr_4 compound.

Figure S18. f_{max} versus 1000/T obtained from the $tan\delta$ plot, showing Arrhenius behavior for (a) Q_2CoBr_4 and (b) Q_2CdBr_4 compound.

Figure S19. EPR spectra of Mn^{2+} recorded for selected temperature range. The narrow lines marked in the figure come from Cr^{3+} ions in the Al_2O_3 crystal, which was permanently placed in the EPR cavity as a standard for the Mn^{2+} spectrum intensity.

Figure S20. Temperature dependence of the EPR Mn^{2+} integral intensity.

Table content:

Table S1. Selected hydrogen bond parameters for Q_2MBr_4 (M = Co, Mn, Cd).

Table S2. The diffraction experimental details for Q_2CdBr_4 in phase II at 100 K.

Table S3. Selected geometric parameters (Å, °) of inorganic polyhedra in Q_2MBr_4 (M = Co, Mn, Cd).

Table S4. The wavenumbers (cm^{-1}) of the observed infrared and Raman bands of Q_2MBr_4 at 300 K.

Figures:

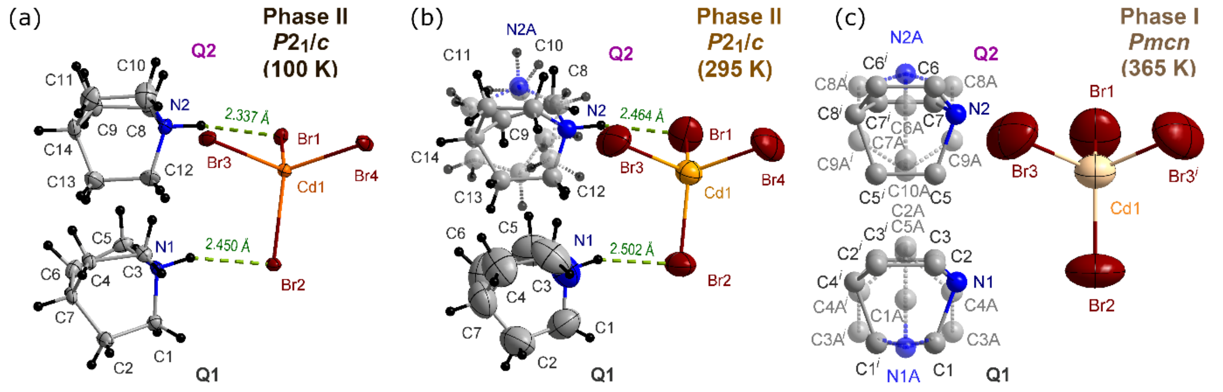

Fig. S1. The asymmetric unit of the Q_2CdBr_4 with atom numbering scheme (a) in phase II at 100 K with frozen motion of Q2 moiety, (b) in phase II at 295 K with disorder of Q2 and (c) in phase I at 365 K with disordered Q1 and Q2 ions (presented in transparent color and with dashed bonds). The symmetry code: (i) $-x+1/2$, y, z. The N—H···Br bonds are presented as green dashed lines.

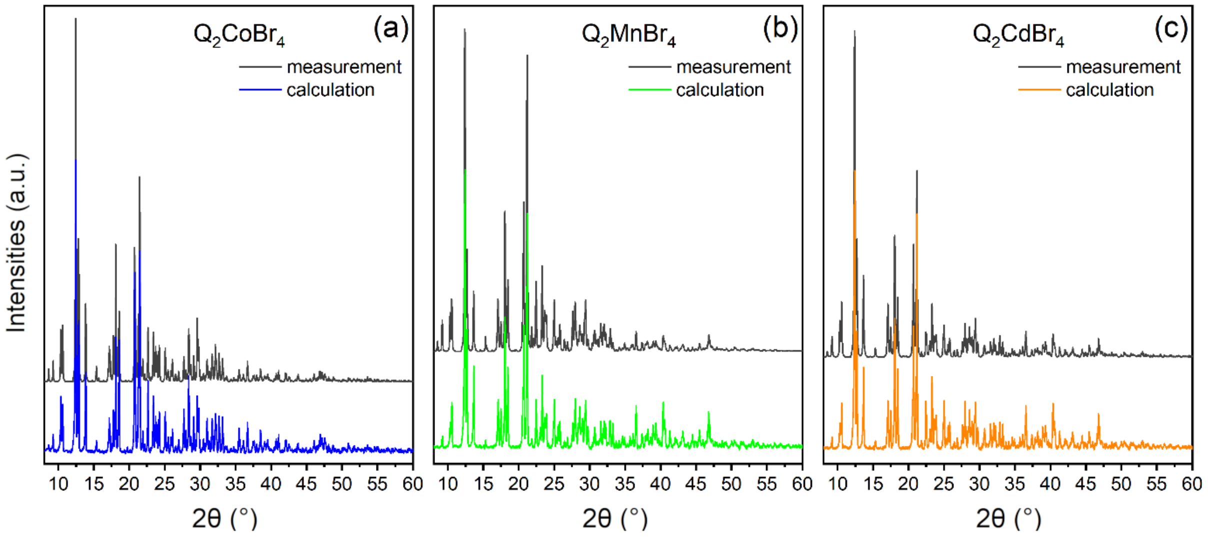

Fig. S2. The powder X-ray diffraction data of studied compounds.

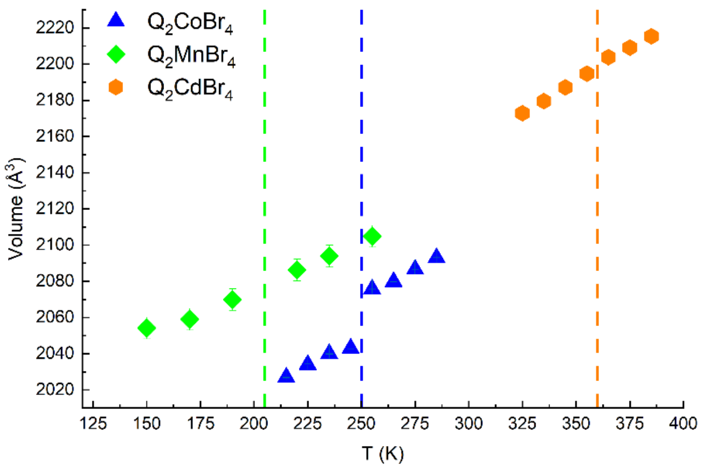

Fig. S3. The volume changes around PT for Q_2CoBr_4, Q_2MnBr_4 and Q_2CdBr_4 derived from X-ray diffraction measurements.

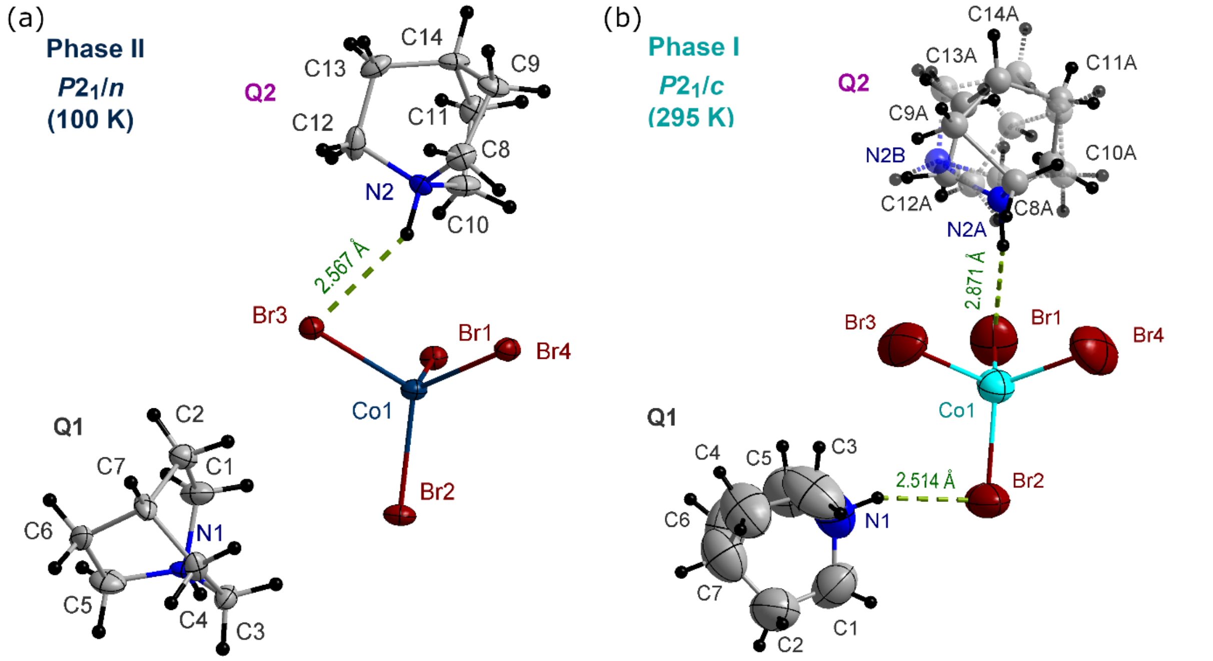

Fig. S4. The asymmetric unit of the Q_2CoBr_4 with atom numbering scheme: (a) ordered phase II at 100 K and (b) phase II at 295 K with disordered Q2 cation (presented in transparent color and with dashed bonds). The N—H···Br bonds are presented as green dashed lines.

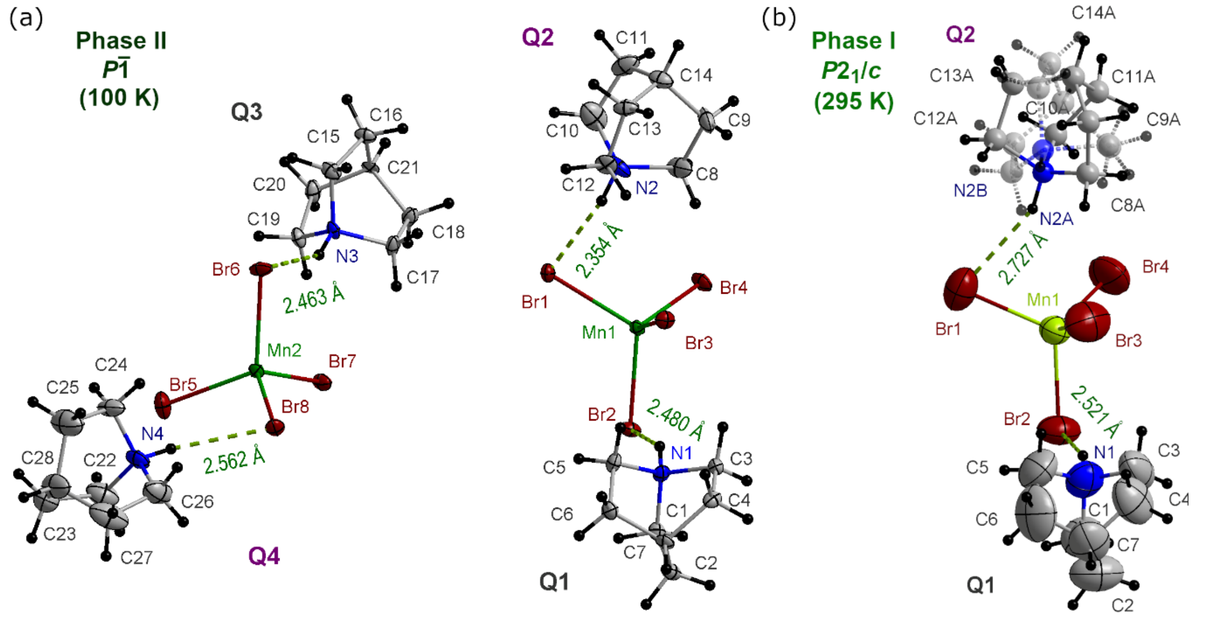

Fig. S5. The asymmetric unit of the Q_2MnBr_4 with atom numbering scheme: (a) ordered phase II at 100 K and (b) phase II at 295 K with disordered Q2 cation (presented in transparent color and with dashed bonds). The N—H···Br bonds are presented as green dashed lines.

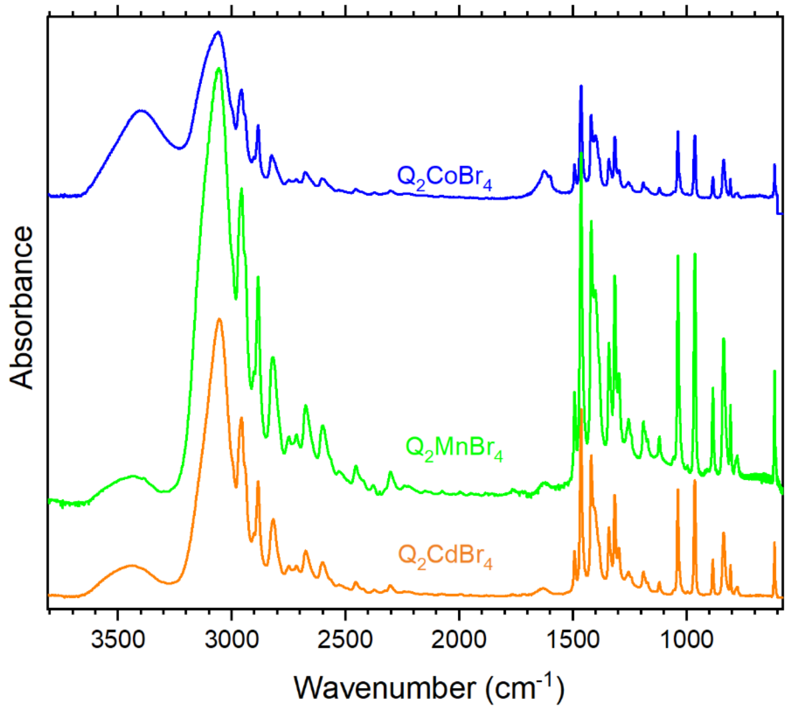

Fig. S6. The RT IR spectra of Q_2MBr_4 crystals.

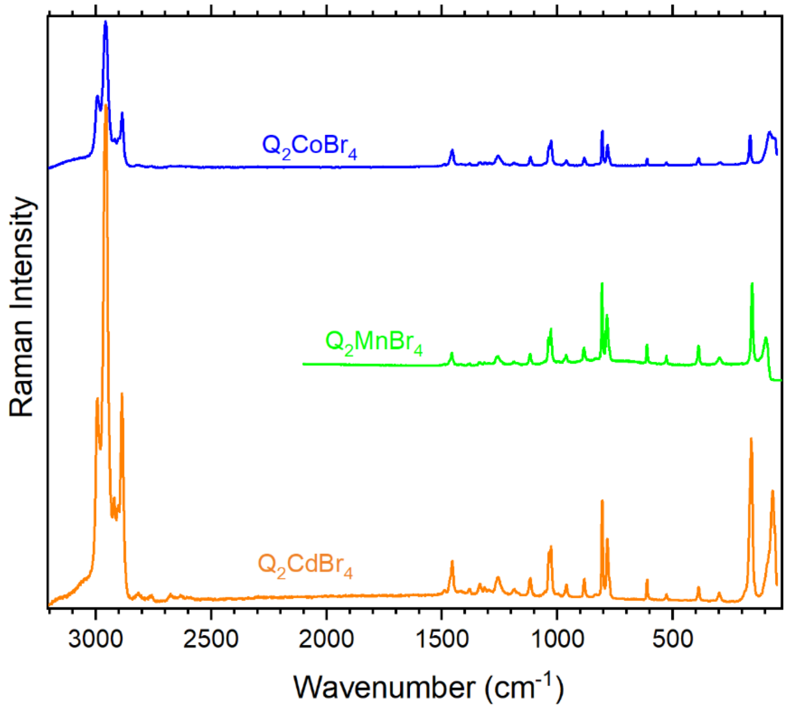

Fig. 7. The RT Raman spectra of Q_2MBr_4.

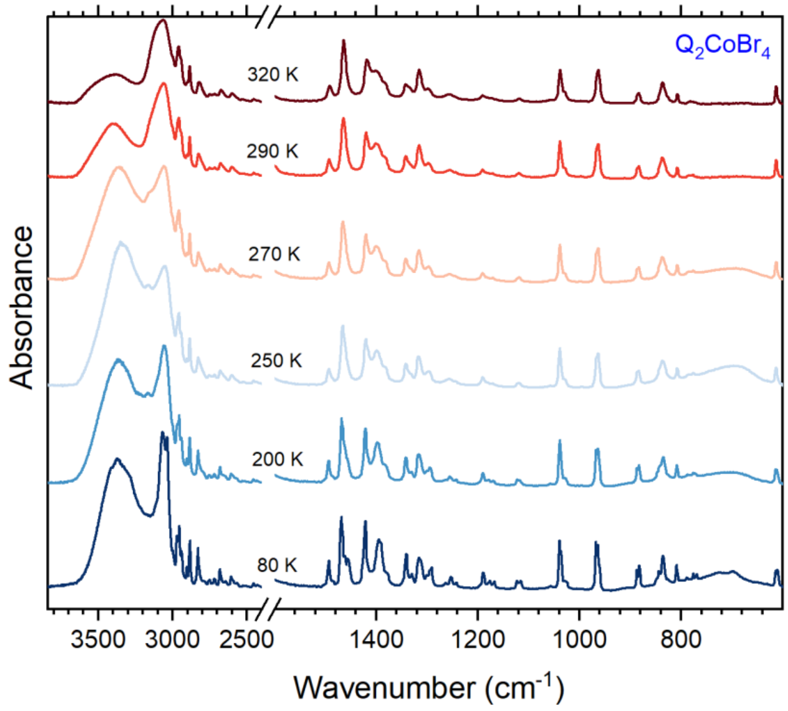

Fig. S8. The temperature-dependent IR spectra of Q_2CoBr_4.

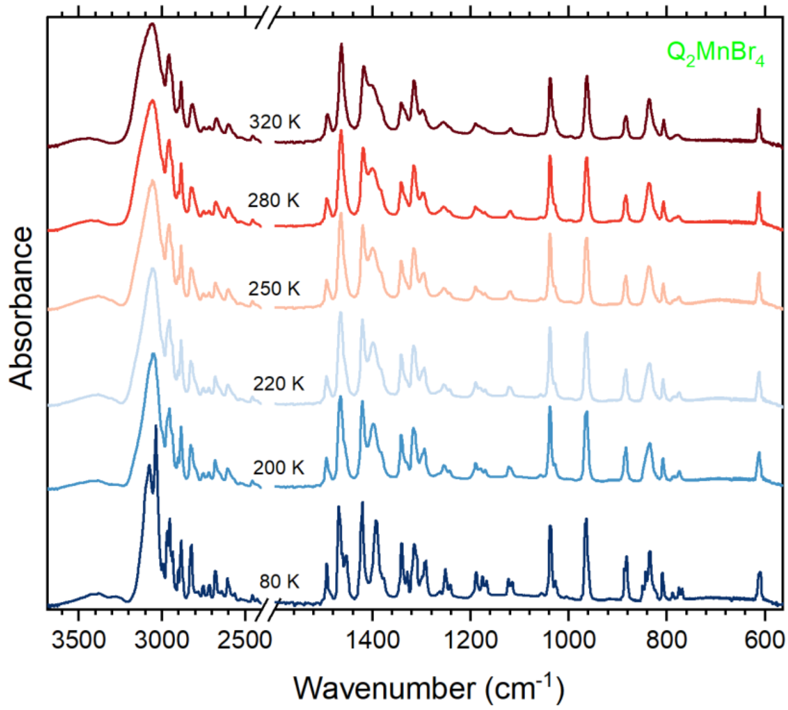

Fig. S9. The temperature-dependent IR spectra of Q_2MnBr_4.

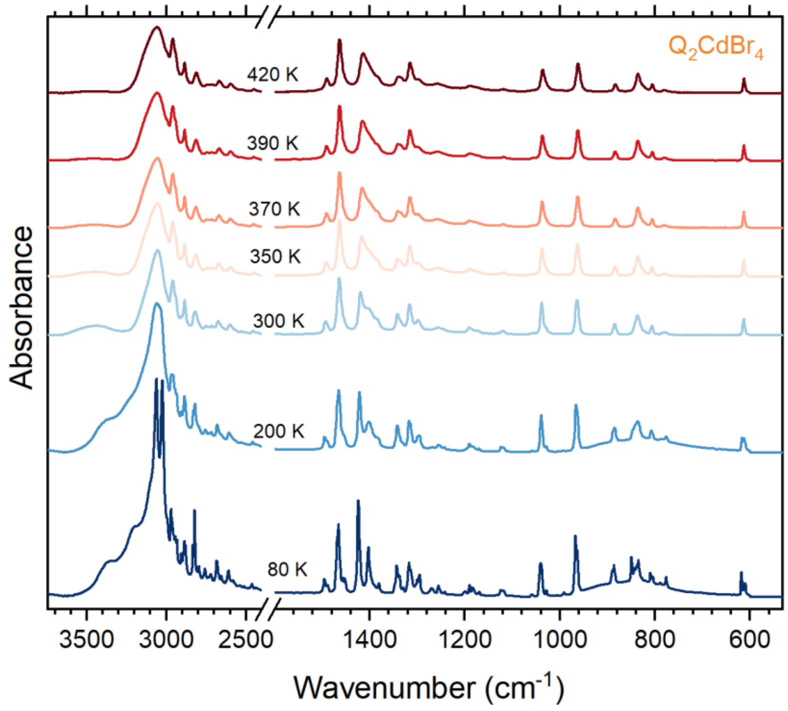

Fig. S10. The temperature-dependent IR spectra of Q_2CdBr_4.

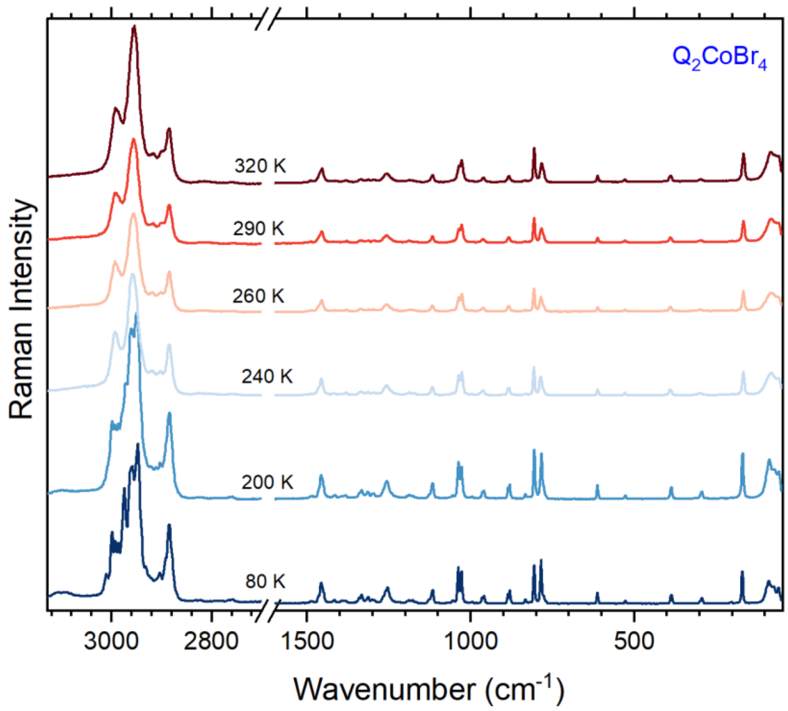

Fig. S11. The temperature-dependent Raman spectra of Q_2CoBr_4.

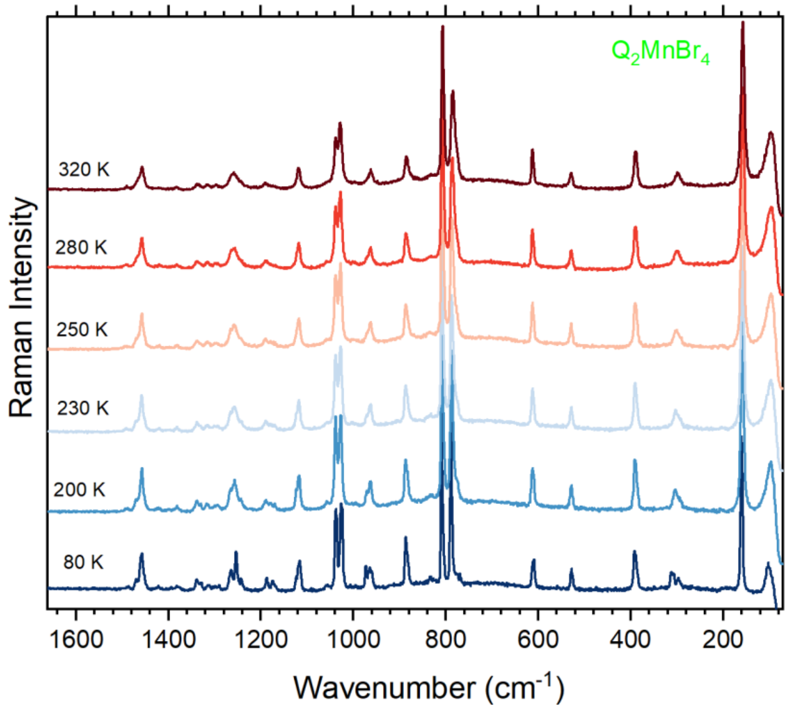

Fig. S12. The temperature-dependent Raman spectra of Q_2MnBr_4.

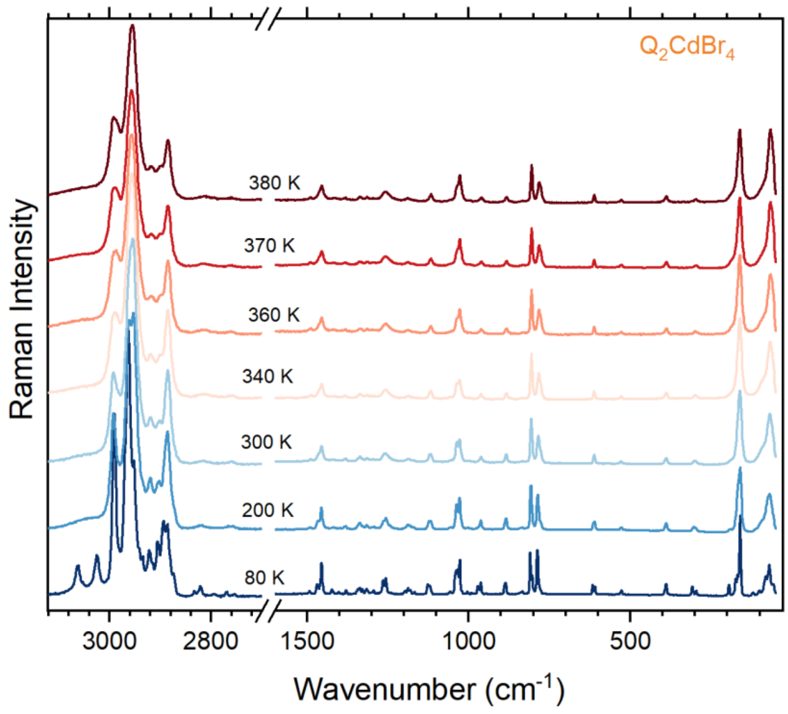

Fig. S13. The temperature-dependent Raman spectra of Q_2CdBr_4.

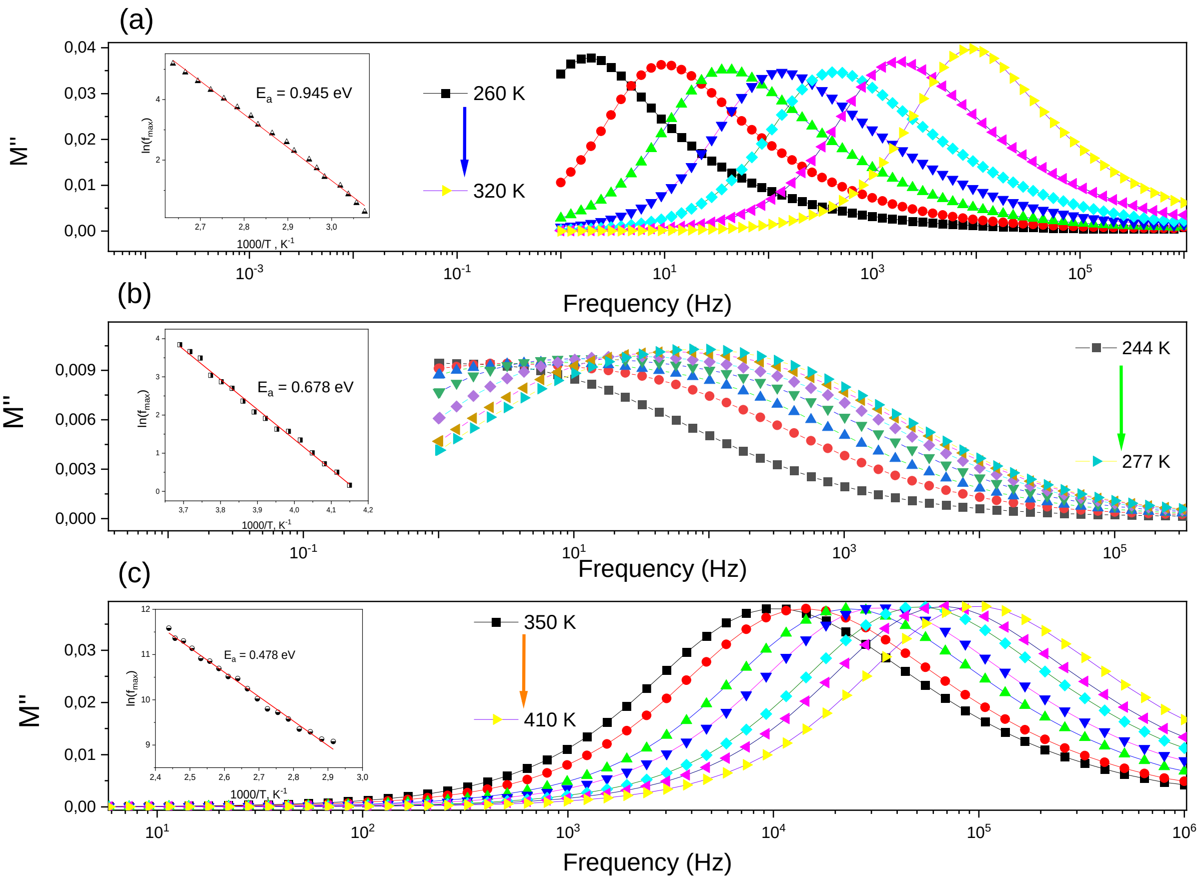

Fig. S14. Variation of imaginary part of the complex electric modulus[M ''(ω)] with frequency (f) for (a) Q_2CoBr_4, (b) Q_2MnBr_4 and (c) Q_2CdBr_4. Insert: ln(f_{max}) versus 1000/T plot obtained from the complex electrical modulus plot showing Arrhenius behavior.

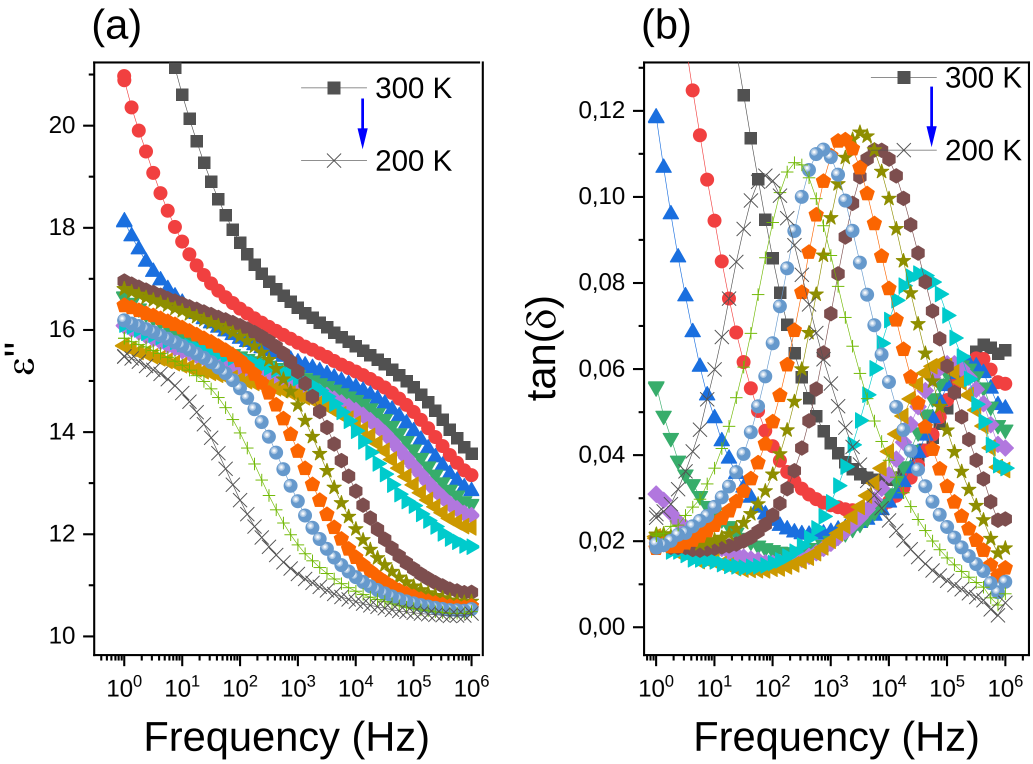

Fig. S15. Frequency-dependent real part of (a) dielectric constant (ε′) and (b)loss tangent (tanδ) at over the temperature range 300–200 K for Q_2CoBr_4 compound.

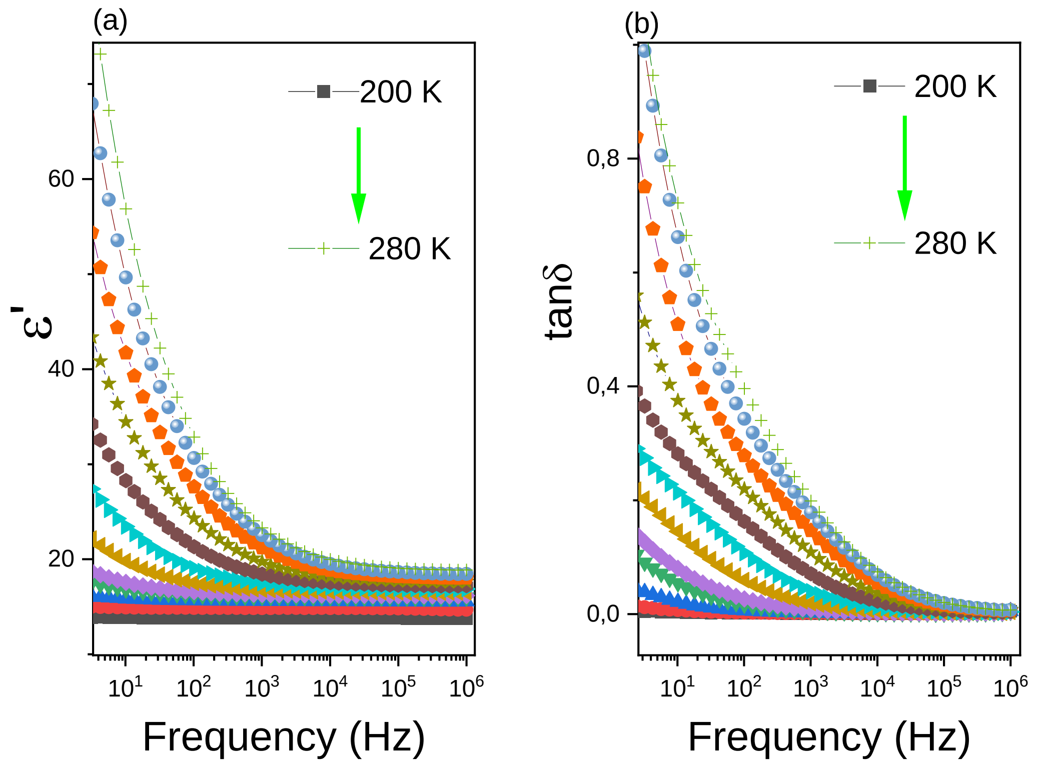

Fig. S16. Frequency-dependent real part of (a) dielectric constant (ε′) and (b)loss tangent (tanδ) at over the temperature range 300–200 K for Q_2MnBr_4 compound.

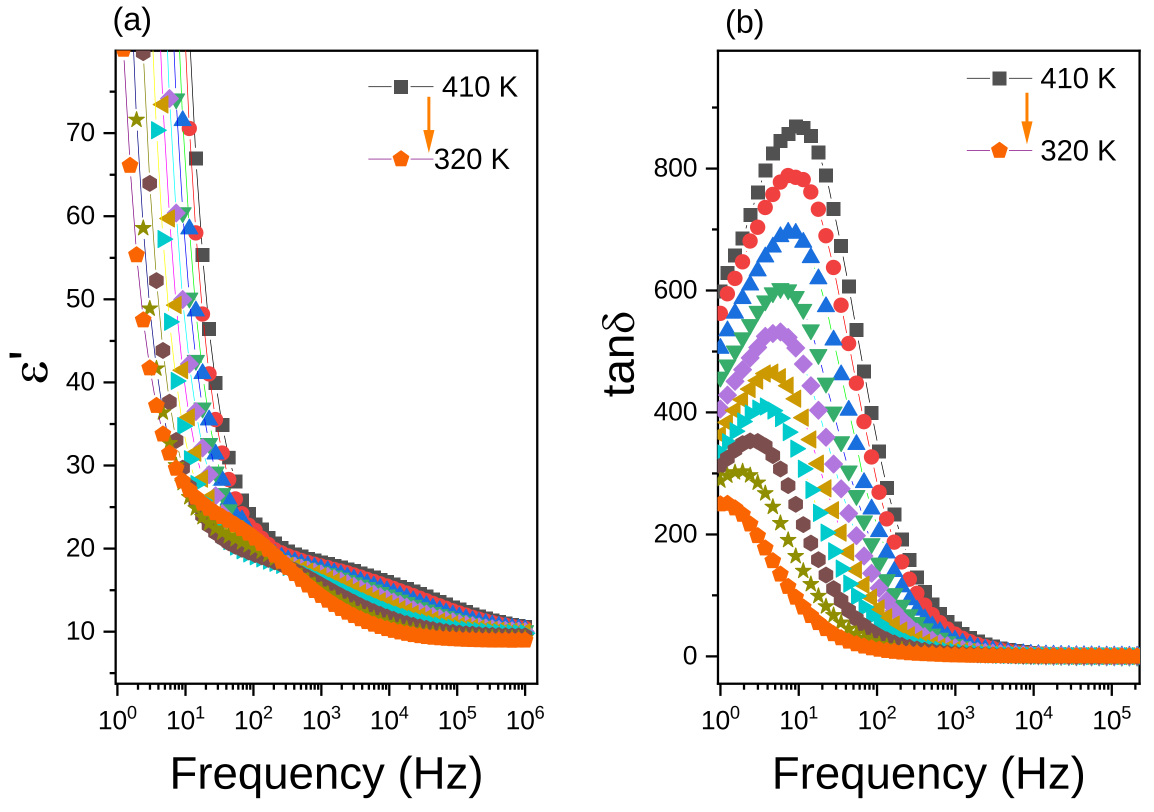

Fig. S17. Frequency-dependent real part of (a) dielectric constant (ε′) and (b)loss tangent (tanδ) at over the temperature range 300–200 K for Q_2CdBr_4 compound.

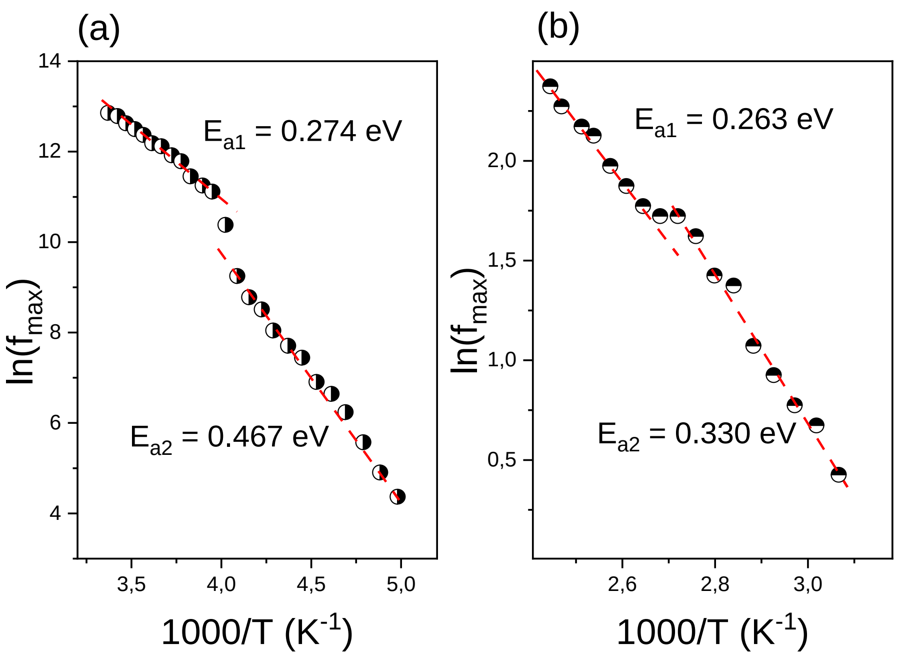

Fig. S18. f_{max} versus 1000/T obtained from the $tan\delta$ plot, showing Arrhenius behavior for (a) Q_2CoBr_4 and (b) Q_2CdBr_4 compound.

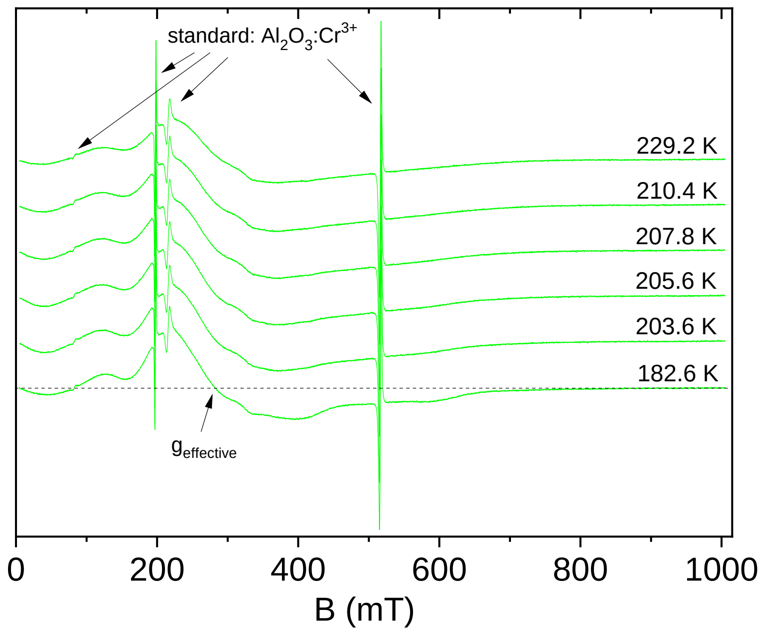

Fig. S19. EPR spectra of Mn^{2+} recorded for selected temperature range. The narrow lines marked in the figure come from Cr^{3+} ions in the Al_2O_3 crystal, which was permanently placed in the EPR cavity as a standard for the Mn^{2+} spectrum intensity.

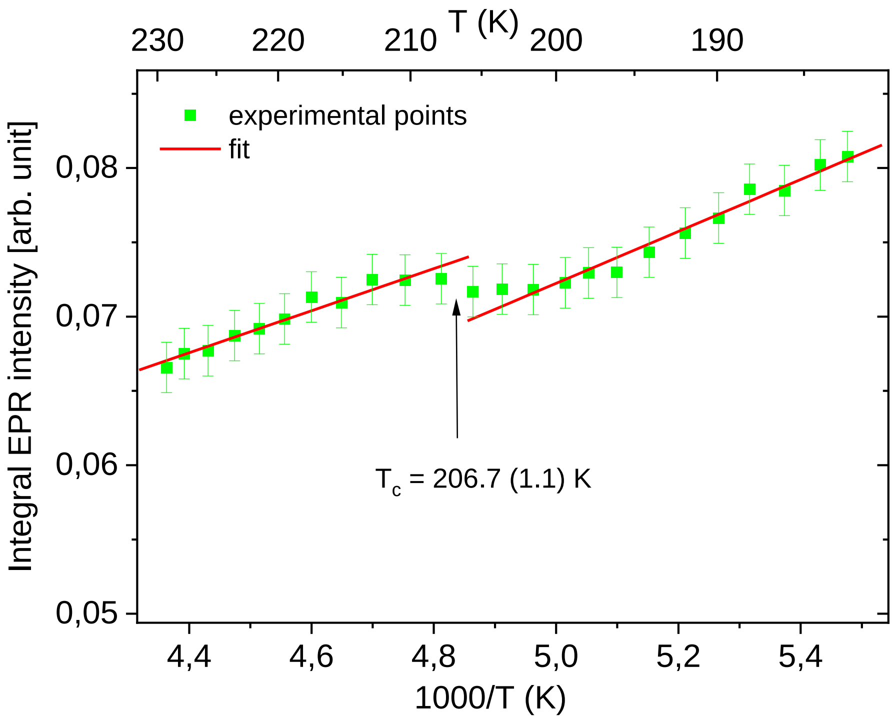

Fig. S20. Temperature dependence of the EPR Mn^{2+} integral intensity.

Tables:

Table S1. Selected hydrogen bond parameters for Q_2MBr_4 (M = Co, Mn, Cd).

		D—H···*A*	*D*—H (Å)	H···*A* (Å)	*D*···*A* (Å)	*D*—H···*A* (°)
Q_2CoBr_4	Phase II 100 K	N1—H1···Br2^{i}	0.98	2.50	3.382(2)	147
		N2—H2···Br3	0.98	2.57	3.444(2)	146
	Phase I 295 K	N1—H1···Br2	0.98	2.51	3.429(8)	155
		N2*A*—H2*AA*···Br1	0.98	2.87	3.685(2)	141
		N2*B*—H2*BA*···Br1^{i}	0.98	2.81	3.57 (3)	136
Q_2MnBr_4	Phase II 100 K	N1—H1···Br2	0.98	2.48	3.359(5)	147
		N2—H2···Br1	0.98	2.35	3.344(5)	171
		N3—H3···Br6	0.98	2.46	3.369(5)	150
		N4—H4···Br8	0.98	2.56	3.443(6)	147
	Phase I 295 K	N1—H1···Br2	0.98	2.52	3.437(8)	156
		N2*A*—H2*AA*···Br1	0.98	2.73	3.640(2)	155

			N2*B*—H2*BA*···Br1[i]	0.98	2.58	3.45 (3)	148
Q_2CdBr_4	Phase II	100 K	N1—H1···Br2	0.98	2.45	3.372(2)	157
			N2—H2···Br1	0.98	2.34	3.284(2)	162
		295 K	N1—H1···Br2	0.98	2.50	3.418(6)	156
			N2—H2···Br1	0.98	2.46	3.43 (2)	167
			N2*A* —H2*AA*···Br1[i]	0.98	2.76	3.641(2)	149

Symmetry code: (i) −*x*+1, −*y*+1, −*z*+1.

Table S2. The diffraction experimental details for Q_2CdBr_4 in phase II at 100 K.

Q_2CdBr_4 Phase II	
Crystal data	
Chemical formula	$2(C_7H_{14}N){\cdot}CdBr_4$
M_r	656.42
Crystal system, space group	Monoclinic, $P2_1/c$ (no. 14)
T (K)	100
a, b, c (Å)	9.636(3), 16.706(4), 12.832(3)
β (°)	91.79(2)
V (Å^3)	2064.7(8)
Z	4
μ (mm^{-1})	8.80
Crystal size (mm)	0.28 × 0.17 × 0.14
Data collection	
Refl. measured/ unique/ observed [$I > 2\sigma(I)$]	12747/ 4211/ 3562
R_{int}	0.027
Refinement	
$R[F^2 > 2\sigma(F^2)]$, $wR(F^2)$, S	0.023, 0.043, 1.03
Data/ parameters/ restraints	4211/ 190/ 0
$\Delta\rho_{max}$, $\Delta\rho_{min}$ (e Å^{-3})	0.53, −0.47

Table S3. Selected geometric parameters (Å, °) of inorganic polyhedra in Q_2MBr_4 (M = Co, Mn, Cd).

Crystal	Phase (temp.)	Bond	Bond length (Å)	Angle description	Angle value (°)
Q_2CoBr_4	Phase II (100 K)	Co1—Br1	2.405(2)	Br1—Co1—Br2	107.27(8)
		Co1—Br2	2.417(2)	Br1—Co1—Br3	108.28(8)
		Co1—Br3	2.420(2)	Br1—Co1—Br4	111.20(9)
		Co1—Br4	2.409(2)	Br2—Co1—Br3	112.79(8)
				Br4—Co1—Br2	112.69(8)

				Br4—Co1—Br3	104.58(8)
	**Phase I (295 K)**	Co1—Br1	2.4082(15)	Br1—Co1—Br3	106.44(6)
		Co1—Br2	2.4040(15)	Br2—Co1—Br1	114.65(5)
		Co1—Br3	2.4107(15)	Br2—Co1—Br3	106.55(6)
		Co1—Br4	2.3894(15)	Br4—Co1—Br1	105.63(6)
				Br4—Co1—Br2	111.06(6)
				Br4—Co1—Br3	112.57(6)
**Q_2MnBr_4**	**Phase II (100 K)**	Mn1—Br1	2.5351(2)	Br2—Mn1—Br1	116.22(4)
		Mn1—Br2	2.4962(2)	Br2—Mn1—Br3	105.88(5)
		Mn1—Br3	2.5231(2)	Br3—Mn1—Br1	103.82(5)
		Mn1—Br4	2.4799(2)	Br4—Mn1—Br1	105.43(5)
		Mn2—Br5	2.5022(2)	Br4—Mn1—Br2	111.06(5)
		Mn2—Br6	2.5103(2)	Br4—Mn1—Br3	114.52(4)
		Mn2—Br7	2.5021(2)	Br6—Mn2—Br5	114.98(5)
		Mn2—Br8	2.5141(2)	Br6—Mn2—Br7	104.42(5)
				Br7—Mn2—Br5	109.36(5)
				Br8—Mn2—Br5	101.44(5)
				Br8—Mn2—Br6	109.96(5)
				Br8—Mn2—Br7	117.13(4)
	Phase I (295 K)	Mn1—Br1	2.5084(15)	Br2—Mn1—Br1	115.15(5)
		Mn1—Br2	2.4982(15)	Br2—Mn1—Br3	105.62(6)
		Mn1—Br3	2.5061(16)	Br3—Mn1—Br1	105.76(6)
		Mn1—Br4	2.4819(16)	Br4—Mn1—Br1	105.54(6)
				Br4—Mn1—Br2	111.52(6)
				Br4—Mn1—Br3	113.33(6)
**Q_2CdBr_4**	**Phase II (100 K)**	Cd1—Br1	2.6097(7)	Br2—Cd1—Br1	112.559(13)
		Cd1—Br2	2.6071(7)	Br2—Cd1—Br3	102.77(2)
		Cd1—Br3	2.6083(6)	Br3—Cd1—Br1	107.191(17)
		Cd1—Br4	2.5516(6)	Br4—Cd1—Br1	107.881(16)
				Br4—Cd1—Br2	112.17(2)
				Br4—Cd1—Br3	114.224(19)
	**Phase II (295 K)**	Cd1—Br1	2.5975(11)	Br2—Cd1—Br1	115.77(3)
		Cd1—Br2	2.5839(11)	Br2—Cd1—Br3	104.42(4)
		Cd1—Br3	2.5921(12)	Br3—Cd1—Br1	105.16(4)
		Cd1—Br4	2.5597(12)	Br4—Cd1—Br1	105.60(4)
				Br4—Cd1—Br2	111.84(4)
				Br4—Cd1—Br3	114.13(4)
	**Phase I (365 K)**	Cd1—Br1	2.581(2)	Br2—Cd1—Br1	114.38(9)
		Cd1—Br2	2.556(2)	Br2—Cd1—Br3	109.49(5)

Cd1—Br3	2.5706(17)	Br3—Cd1—Br1	105.78(6)
Cd1—Br3^{i}	2.5706(17)	Br3^{i}—Cd1—Br1	105.78(6)
		Br3^{i} —Cd1—Br2	109.49(5)
		Br3^{i}—Cd1—Br3	111.87(10)

Table S4. The wavenumbers (cm^{-1}) of the observed infrared and Raman bands of Q_2MBr_4 at 300 K.

Q_2CoBr_4		Q_2MnBr_4		Q_2CdBr_4		Assignment
**IR**	**Raman**	**IR**	**Raman**	**IR**	**Raman**	
3400m		3442m		3437m		νNH
3061vs		3058vs		3052vs		νNH νCH $\nu_{as}CH_2$
	2992s				2993s	$\nu_{as}CH_2$
2959s	2956vs	2957s		2958s	2955vs	$\nu_{as}CH_2$ ν_sCH_2
2943sh		2941sh		2943sh		$\nu_{as}CH_2$
	2918m				2919m	ν_sCH_2
2903w	2901m	2901w		2904w	2902m	ν_sCH_2
2885m	2885s	2885m		2885m	2885s	$\nu_{as}CH_2$ ν_sCH_2
2826m		2820m		2816m		overtone
2678w		2673w		2675w		combination
2602w		2601w		2601w		combination
1628w		1626vw		1630vw		γNH
1600sh						γNH
1493w		1493w		1493w		δCH_2
1463s		1464s		1464s		δCH_2
	1456w		1458w		1454w	δCH_2
1420s		1419s		1419s		γNH δCH_2
1401w		1402w		1403w		γNH δCH_2
1382sh	1379vw			1384sh	1382vw	δCH_2
1343w	1337vw	1342w	1338vw	1340w	1336vw	ωCH_2
1314m		1317m		1317m	1315vw	γCH ωCH_2
1297w	1298vw	1298w	1298vw	1300w	1297vw	ωCH_2
1256w	1257w	1256w	1261vw	1255w	1257w	τCH_2
1190w	1188vw	1190w	1189vw	1190w	1188vw	τCH_2
1119w	1118vw	1120w	1119vw	1118w	1118vw	$\nu_{as}CNC$
1038m	1038w	1039m	1039w	1039m	1036w	$\nu_{as}CNC$ $\nu_{as}CCC$
	1028w		1028w		1028w	ν_sCNC ν_sCCC
964m	962vw	964m	962vw	964m	962vw	ν_sCNC ν_sCCC τCH_2
885w	885vw	884w	887vw	884w	883vw	γNH
837w		837w		837w		γNH

807w	806m	807w	807m	807w	806m	ν_sCNC ν_sCCC
780vw	784w	779vw	786w	779vw	784w	ν_sCNC ν_sCCC
612w	613w	613w	613w	613w	612w	δCNC δCCC
	529vw		529vw		528vw	δCNC δCCN
	390w		390w		400w	δCNC δCCN
	296vw		299vw		298w	$\nu_{as}MBr_4$ T'Q
	166s		158s		161s	ν_sMBr_4 T'Q
	81m		96m		68m	δMBr_4 LQ

Key: s-very strong, s-strong, m-medium, w-weak, vw-very weak, sh-shoulder; ν_s–symmetric stretching, ν_{as}–asymmetric stretching, δ-in-plane bending, ρ-rocking, ω-wagging, τ-twisting (torsion), γ-out-of-plane bending, T–translation, L–libration.
